# Outwitting EF-Tu and the ribosome: translation with d-amino acids

**DOI:** 10.1093/nar/gkv566

**Published:** 2015-05-30

**Authors:** John Achenbach, Michael Jahnz, Lucas Bethge, Krisztina Paal, Maria Jung, Maja Schuster, Renate Albrecht, Florian Jarosch, Knud H. Nierhaus, Sven Klussmann

**Affiliations:** 1 NOXXON Pharma AG, Max-Dohrn-Strasse 8–10, 10589 Berlin, Germany; 2 Institut für Medizinische Physik und Biophysik, Charité – Universitätsmedizin Berlin, Charitéplatz 1, 10117 Berlin, Germany

## Abstract

Key components of the translational apparatus, i.e. ribosomes, elongation factor EF-Tu and most aminoacyl-tRNA synthetases, are stereoselective and prevent incorporation of d-amino acids (d-aa) into polypeptides. The rare appearance of d-aa in natural polypeptides arises from post-translational modifications or non-ribosomal synthesis. We introduce an *in vitro* translation system that enables single incorporation of 17 out of 18 tested d-aa into a polypeptide; incorporation of two or three successive d-aa was also observed in several cases. The system consists of wild-type components and d-aa are introduced via artificially charged, unmodified tRNA^Gly^ that was selected according to the rules of ‘thermodynamic compensation’. The results reveal an unexpected plasticity of the ribosomal peptidyltransferase center and thus shed new light on the mechanism of chiral discrimination during translation. Furthermore, ribosomal incorporation of d-aa into polypeptides may greatly expand the armamentarium of *in vitro* translation towards the identification of peptides and proteins with new properties and functions.

## INTRODUCTION

Naturally occurring nucleic acids are composed of nucleotides in the d-configuration, whereas proteins almost exclusively consist of amino acids in the l-configuration, a phenomenon known as the homochirality of life. Nevertheless, amino acids in the d-configuration also play an important role in all kingdoms of life. They are found, e.g. in bacterial cell walls, antibiotics, peptide hormones or animal venoms; in most cases, there is one d-aa per peptide chain (1,2). For example, deltorphins are heptameric peptides isolated from the skin of south American tree frogs belonging to the subfamily *Phyllomedusinae*, which contain either d-Ala, d-Leu or d-Met in position 2 and have been shown to be potent opioids, whereas the corresponding all-l-peptides are virtually inactive (1,3). A single d-aa in a peptide can thus be the game changer. d-amino acids may enhance the folding space, adding structural features that cannot be constituted by l-amino acids (l-aa) alone. In this respect, d-aa have recently been shown to improve both the stability and target inhibition of peptide-ligands dedicated for pharmaceutical applications (4).

In nature, the rare appearance of d-aa in polypeptides originates either from non-ribosomal synthesis (2) or post-translational isomerization (1), they are not incorporated by ribosomal synthesis. Since the accidental incorporation of a d-aa into a nascent protein may hamper the folding into its native conformation, a high evolutionary selection pressure on chiral discrimination during the translation process can be assumed. Indeed, key components of the translational apparatus show a strong stereoselectivity at three levels: first, most aminoacyl-tRNA synthetases (aaRS) charge tRNAs exclusively with l-aa (5,6), second, elongation factor EF-Tu that shuttles aminoacyl-tRNAs (aa-tRNAs) to the ribosome in ternary complexes (TCs; aa-tRNA•EF-Tu•GTP) discriminates against d-aa-tRNAs (7,8) and finally the ribosome that rejects d-aa-tRNAs (7,9–12). In this way, a strong defence against the accidental incorporation of d-aa is established. The overall stereoselectivity of the translational apparatus has been calculated to be 3 × 10^4^ for d-tyrosine (factor 25 for the aminoacylation step × 250 for EF-Tu × 5 for the ribosome) (7). Taking into account that TyrRS is permissive for d-Tyr (5,13), this factor is likely to be higher for other d-aa that are already rejected during the aminoacylation step. This discrimination factor also compares well with the value determined for the misincorporation of l-amino acids that occurs at a rate of one in ≈10^3^–10^4^ (reviewed in (14)). After techniques to rewire the genetic code to non-canonical amino acids became available (15), a plethora of those amino acids have been incorporated into polypeptides by ribosomal translation (for review, see (16)). However, the incorporation of d-aa has been attempted many times with disappointing results and it still poses a challenge (17–23). The efforts gained momentum when the Hecht group demonstrated significant incorporation of two d-aa (d-Phe and d-Met) using mutant ribosomes (23S rRNA 2447–2450 GAUA to UGGC), which was not possible with wild-type ribosomes (11). However, these ribosomes have the drawback of an impaired accuracy (24) and the mutations are associated with a recessive-lethal phenotype (25,26). Later, the Suga group demonstrated initiation of ribosomal peptide synthesis using d-amino acids so that the d-amino acid position is restricted to the N-terminus of a peptide (27). Initiation is mechanistically very different from incorporation at any other site by elongation and does not involve EF-Tu and the elements of the peptidyl-transferase center belonging to the ribosomal A-site, which are important chiral discriminators. The same study showed that d-Cys and d-Met could not be incorporated by translational elongation. Here, the d-amino acids were delivered by an amber-suppressor tRNA, tRNA^Asn-E2^_CUA_. Subsequently, the same group also achieved single incorporation of 12 different d-amino acids using essentially the same experimental setup, changing only the anticodon of this tRNA to a serine-specific anticodon and the respective codon on the mRNA. However, seven d-aa (Arg, Asp, Lys, Glu, Ile, Pro, Trp) remained resistant to translational elongation, which the authors attributed to the stereoselectivity of EF-Tu (28).

We tackled the task of d-aa incorporation by overcoming the deleterious effect of chiral discrimination against d-aa at all three levels using a defined, reconstituted *in vitro* translation system comprising native factors and ribosomes and succeeded in finding the key to enabling the incorporation of 17 different d-aa.

## MATERIALS AND METHODS

### Ribosome purification

Ribosomes were prepared from *Escherichia coli* strain Can20–12e (29) as described earlier (30).

### Cloning, expression and purification of recombinant proteins

A detailed description is given in the Supplementary Information.

### Synthesis of Flexizyme substrates

A detailed description of the synthesis strategy along with nuclear magnetic resonance data and enantiopurities of d-aa Flexizyme substrates is given in the Supplementary Information. The enantiopurity was determined by C.A.T. GmbH using a well-established chiral gas chromatography–mass spectrometry (GC-MS) technique (31).

### Radiolabeling of tRNA

3′-[^32^P]-Radiolabeling of tRNA using tRNA nucleotidyltransferase was carried out following a published protocol (32) with minor modifications as given in the Supplementary Information.

### Misacylation of tRNA

tRNA was misacylated using Flexizymes following a previously published protocol (33) with modifications. A detailed description is provided in the Supplementary Information.

### EF-Tu electrophoretic mobility shift assay

The electrophoretic mobility shift assay (EMSA) assay was designed following earlier examples (34,35). To set the GTP state, EF-Tu was first incubated with GTP in the presence of phosphoenol pyruvate and pyruvate kinase in a mastermix, which was then spread into reaction vessels containing 3′-^32^P radiolabeled, misacylated tRNA. Incubation was resumed for TC formation and the reactions were subjected to native PAGE. TCs and free tRNA were detected and quantified by phosphorimaging. Since only aminoacyl-tRNA can be bound by EF-Tu•GTP, the aminoacylation ratio of each batch of aa-tRNA was determined following (32); only aspartic acid was elusive in this assay. The data shown in Figure 2 were calculated as [(TC-Signal) / (free tRNA signal)]/(aminoacylation ratio). For details, see Supplementary Information.

**Figure 1.**
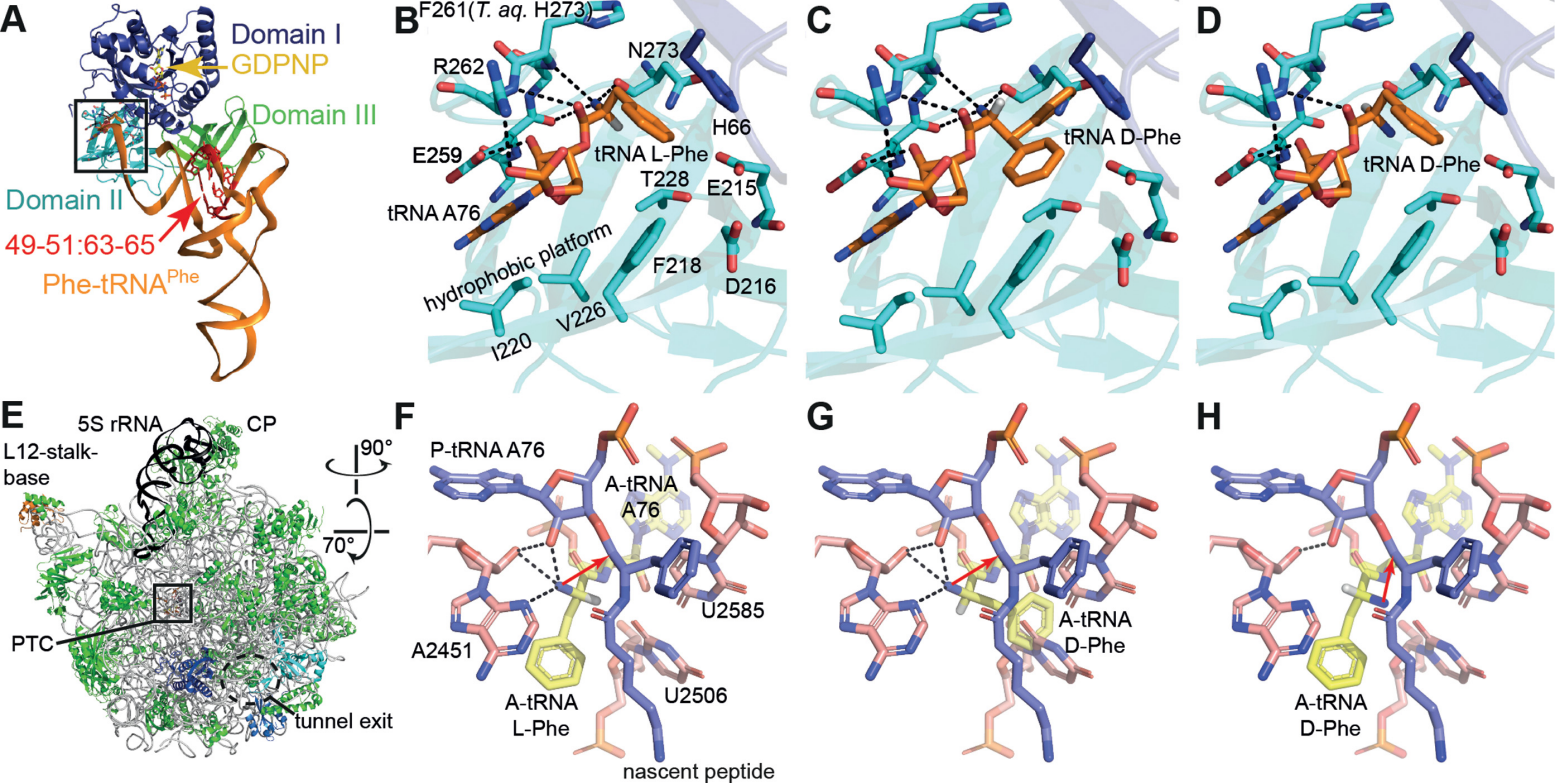
l-aa-tRNA and d-aa-tRNA bound to EF-Tu and the ribosomal A-site. (**A**) Structure of a TC l-Phe-tRNA^Phe^-EF-Tu-GDPNP. (**B**) Zoom into the EF-Tu amino acid binding pocket. Hydrogen bonds are indicated as dashed lines. (**C, D**) Models of d-Phe-tRNA^Phe^ bound to EF-Tu. In (C), the d-Phe side chain faces steric constraints or clashes within the pocket, whereas in (D), clashes are avoided but interactions between EF-Tu and the d-Phe α-amino group are lost. (**E**) Cytosol side or backside view of the 50S ribosomal subunit. (**F**) the ribosomal PTC with an l-Phe-tRNA in the A-site. The α-amino group makes several interactions and starts a nucleophilic attack on the peptidyl-tRNA ester carbon for peptide bond formation (red arrow). (**G**,**H**) Models of d-Phe-tRNA in the A-site. In (G), the side chain clashes with U2585 and U2506, whereas in (H), the side chain can be accommodated but the α-amino group is turned into an unfavorable position for peptidyl transfer. Coordinates from PDBs 1TTT (41) and 1VQN (45).

**Figure 2.**
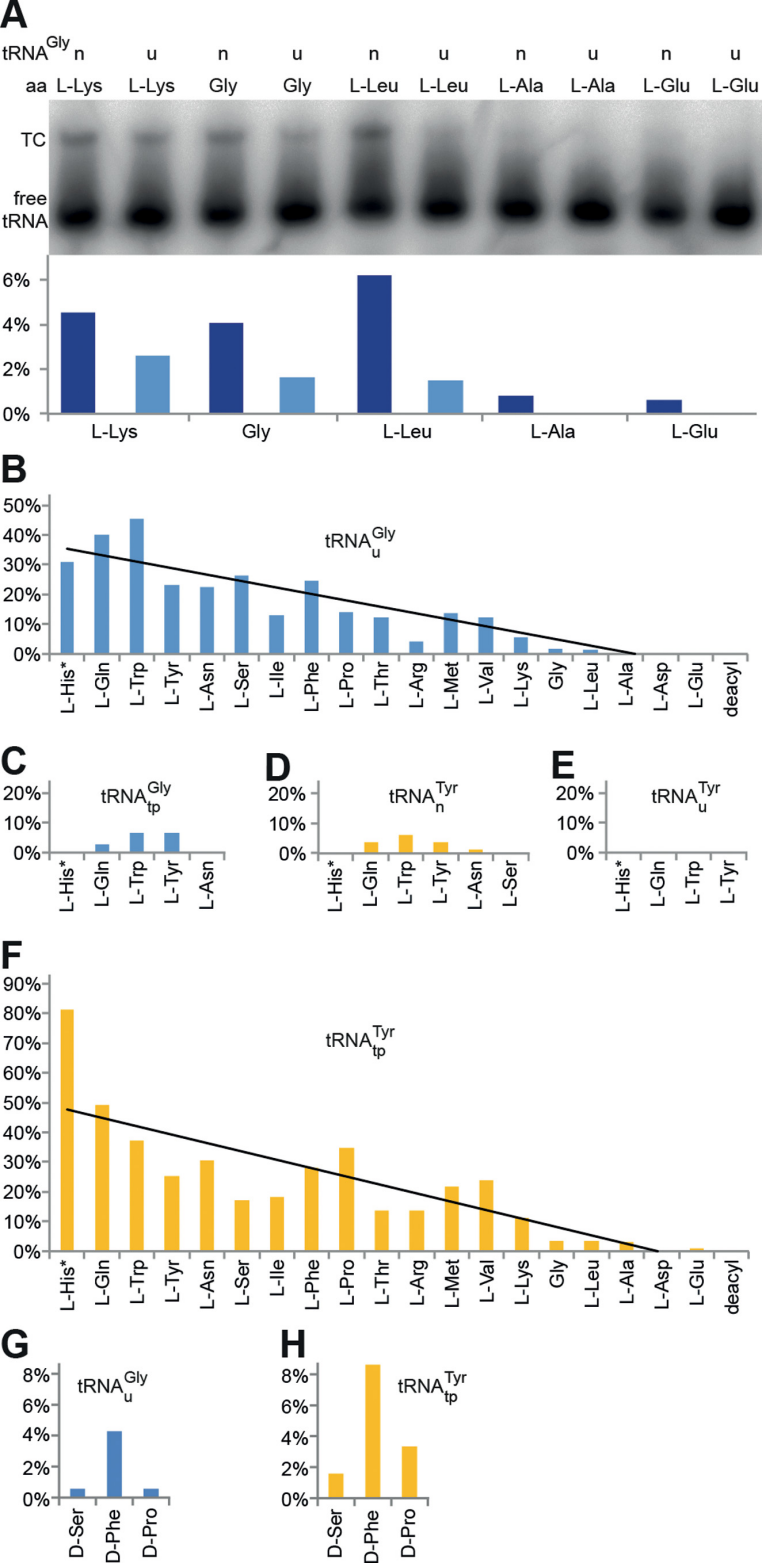
TC formation with wild-type EF-Tu•GTP and aa-tRNA. The esterified amino acids are ordered in a descending order of binding energy contribution according to (47) (* data for l-His were unavailable, we arbitrarily put it to the left). (**A**) Upper panel: EMSA with native (n) and unmodified (u) tRNA^Gly^ acylated with weakly contributing l-aa. Lower panel: relative quantification of TCs versus unbound tRNA. (**B–F**) Evaluation of EMSAs with several tRNAs acylated with canonical aa. (B) unmodified tRNA^Gly^_u_, (C) transplanted tRNA^Gly^_tp_, (D) native tRNA^Tyr^_n_, (E) unmodified tRNA^Tyr^_u_, (F) transplanted tRNA^Tyr^_tp_. (**G**, **H**) Evaluation of EMSAs with tRNA^Gly^_u_ (G) and tRNA^Tyr^_tp_ (H) misacylated with d-aa (see also Supplementary Figure S1).

### Assembly of the translation system

The cell-free, reconstituted translation system used here is a customized, more modularized hybrid of the PURE (protein synthesis using recombinant elements) system (36) (concerning final concentrations of ribosomes and proteins) and the FIT (flexible *in vitro*translation) system (33) (concerning the translation buffer) and consists of the following modules: Solution 1 contains buffer salts (no magnesium), nucleotides, a formyl- and an energy-donor and deacylated bulk tRNA. Solution 2 is a mix of l-aa, containing only those which are required for the intended purpose. Solution 3 contains ribosomes, initiation factors, elongation factors EF-G and EF-Ts (not EF-Tu), release factors RF1 and RF3 (not RF2), ribosome recycling factor (RRF) and energy recycling enzymes. Solution 4 contains the aaRS required for the intended purpose. Magnesium and EF-Tu were kept separate for more flexibility. The final Mg^2+^ concentration used is 13 mM in presence of 6 mM nucleoside triphosphates, corresponding to a free Mg^2+^ concentration of 4 mM. A detailed description is provided in the Supplementary Information.

### Translation templates

The translation templates are plasmids produced by gene synthesis (IDT); the sequences are given in the Supplementary Information. The mRNA transcripts encode the peptide sequence fMSKAKFARTKPHANA[x]HHHHHH, whereby [x] marks the designated misincorporation site that consists of either one, two or three consecutive glycine-codons (templates designated ‘G_1_’, ‘G_2_’ and ‘G_3_’, respectively) or an opal stop codon (template ‘O’).

### Activity test of EF-Tu mutants by GFP translation

Translation reactions were prepared from a mastermix containing a plasmid encoding Emerald GFP (**pRSET/EmGFP**, Invitrogen) and the full translation system except EF-Tu (all canonical amino acids, all aaRS). Wild-type or mutant EF-Tu were added to individual reactions and GFP fluorescence was monitored until a plateau was reached. Details are given in the Supplementary Information.

### Translation and detection of ^35^S-Met labeled peptides

Translation reactions were assembled as a mastermix that contains ^35^S-Met and the l-aa and aaRS required to serve all encoded codons of the translation templates (‘G_1_’, ‘G_2_’, ‘G_3_’, ‘O’) except for the codon dedicated as the ‘misincorporation site’. The mastermix was spread to vessels that contained either preacylated tRNA (for l-aa and d-aa reactions) or an equal amount of deacyl-tRNA and also the respective l-aa-tRNA diluted 1:100 (controls). Translation was allowed to proceed for 3 h, the translation products were purified via the C-terminal His-Tag using Ni-NTA agarose and analyzed by tricine sodium dodecyl sulphate-polyacrylamide gel electrophoresis (SDS-PAGE) and phosphorimaging. Details are given in the Supplementary Information.

### Image processing

For a clear visualization of bands, all autoradiograms shown in this work were gamma-reduced using the Image Lab analysis software (Bio-Rad). All image manipulations were applied globally to the entire respective image. Evaluation of autoradiograms was done using original, unaltered data.

### Diastereomeric peptide separation method and mass spectrometric detection

Template ‘G_1_’ was translated in the presence of either deacyl-tRNA^Gly^ (control), l-Trp-tRNA^Gly^ or d-Trp-tRNA^Gly^ and purified using Ni-NTA agarose. Peptides corresponding to the expected full-length products fMSKAKFARTKPHANA[l-W]HHHHHH (‘l-Trp-peptide’) and fMSKAKFARTKPHANA[d-W]HHHHHH (‘d-Trp-peptide’) were chemically synthesized. The diastereomeric peptides were separated by reversed phase HPLC and detected by an online coupled quadrupole mass spectrometer (qTOF) with electrospray injection, thus enabling retention-time-resolved detection by mass. The chemically synthesized peptides were used to establish the method, as standards and for sample spiking. The synthetic tripeptide VYV was added to all samples as an internal standard. Details are given in the Supplementary Information.

## RESULTS

### Aminoacylating d-aa

aaRS, which ligate individual l-amino acids with their native tRNA to form aminoacyl-tRNAs, represent the first hurdle. The aaRS are specific for both of their substrates (tRNA and l-amino acid) and most aaRS do not recognize the corresponding d-aa as substrates. Some aaRS can even deacylate erroneously acylated d-aa via their editing domain. Nevertheless, at least five aaRS (specific for aspartic acid, histidine, lysine, tryptophan and tyrosine) are able to charge d-aa at a reduced yet substantial catalytic rate (5,6). *In vivo* or in lysate-based *in vitro* translation systems, resulting d-aa-tRNAs can then be deacylated by d-Tyr-tRNA^Tyr^ deacylase, an enzyme that selectively deacylates several d-amino acids from tRNA (5). To produce d-aa-tRNA, we bypass the aaRS and instead use ribozymes (so-called Flexizymes) developed by the Suga group that can charge any tRNA with any l-aa or d-aa (33). For this purpose, we chemically synthesized these Flexizymes and their substrates, i.e. d- and l-amino acids esterified with a leaving group. A very high enantiomeric purity of the d-aa-derivatives is considered essential to generate meaningful data. We set the threshold to a purity of >99%, which reflects the specification of commercially available d-aa. The enantiomeric purity of each d-aa derivative was determined by chiral GC-MS and exceeded 99% in all cases. Using Flexizymes, we were able to charge tRNAs with almost any d- and l-aa and thus the first stereoselectivity hurdle could be cleared. The only exception was d-Cys, which we therefore had to omit from this study.

### A quest for the d-aa shuttle

To take the second and third hurdle, a tRNA is required that enables not only sufficiently stable TC formation even when misacylated with a d-aa, but also incorporation of the respective d-aa into a nascent peptide chain at the ribosome. Regarding TC formation with EF-Tu•GTP, the second stereoselectivity hurdle, the following points should be considered: EF-Tu binds native l-aa-tRNAs with high affinities in a narrow *K_d_* range (37,38). Further studies revealed that tRNA and aminoacyl residue add independently to the overall binding energy, whereby the thermodynamic contributions of tRNAs and l-aminoacyl moieties span a range of up to 3.6 and at least 2.5 kcal/mol, respectively (39). Cognate pairs of tRNAs and amino acids have evolved in a way that a tRNA with a high affinity contribution is charged with a weakly contributing amino acid and vice versa, giving rise to near uniform binding energies of approximately −10 kcal/mol, a phenomenon termed thermodynamic compensation (39,40). EF-Tu interacts with an aminoacyl-tRNA mainly at two regions: (i) the elbow region, particularly the TΨC stem-loop, and (ii) backbone atoms of the acceptor stem carrying an amino acid at the 3′-end, which is accommodated in the amino acid binding pocket of EF-Tu (41–43). The α-amino group of the aminoacyl-moiety is bound via a tetrahedral hydrogen bond network, and also the carbonyl-oxygen is bound by hydrogen bonds (41), thereby fixing the orientation of the first atom in the side chain (β-carbon). The amino acid binding pocket is shaped in a way that all canonical l-amino acids can easily be accommodated (41) (Figure 1A and B). We modeled how a d-aa-tRNA might bind to EF-Tu and found that the side chains of d-aa will likely encounter spatial constraints (Figure 1C). This might force the d-aa into an orientation where the side chain can be accommodated, but hydrogen bonds between the d-aa's α-amino group and the binding pocket are disrupted (Figure 1D). According to our model, at least for d-Phe-tRNA binding seems possible even when the interactions between the binding pocket and the amino acid's main chain are maintained. EF-Tu shows a 24-fold decreased affinity for d-Tyr-tRNA^Tyr^ as compared to l-Tyr-tRNA^Tyr^ (*K_d_* 1.2 μM and 50 nM, respectively) (7), thus d-aa-tRNA binding is not completely blocked, but d-Tyr and presumably d-aa in general contribute very little to the overall binding energy. Consequently, we decided to use a tRNA with a high affinity for EF-Tu to compensate.

The third hurdle is the ribosome itself. Before peptide bond formation, in which residues of the peptidyltransferase center (PTC) actively participate, the α-amino group of the l-aminoacyl-moiety of the A-site tRNA is fixed by several hydrogen bonds and commences a nucleophilic attack on the ester carbon of the peptidyl-tRNA (Figure 1F) (44,45). Ribosomal stereoselectivity is believed to arise from steric constraints that a d-aa experiences in the PTC (Figure 1G), so that it may be forced into an unfavorable orientation for peptide bond formation (9,10,12) (Figure 1H). In other words, the forward reaction rate is decreased and a d-aa-tRNA might dissociate from the ribosome before peptide bond formation occurs (7). The configuration for a d-aa depicted in Figure 1H offers less stability due to a reduced network of hydrogen bonds. Nevertheless, the lack of steric clashes might eventually allow peptide bond formation. Whereas all native l-aa-tRNAs stably bind to the ribosome, some tRNAs show a strongly increased dissociation rate if base modifications are lacking (e.g. tRNA^Glu^) or if an esterified amino acid is lacking (e.g. tRNA^Phe^ and tRNA^Tyr^) (46).

In order to establish a robust test system, a tRNA is preferred that is readily available through (large-scale) chemical synthesis. Thus, it should be an unmodified tRNA. In addition, this tRNA not only has to show strong binding to EF-Tu but also stable binding to the ribosomal A-site even in the absence of base modifications or an esterified amino acid, since we anticipate that the d-aa might fail to aid a tRNA in ribosome binding due to the reduced hydrogen bond network. In the available literature, we find these properties combined only in tRNA^Gly^ (46,47) and it is therefore our prime candidate for the incorporation of d-aa.

### TC formation with d-aa-tRNA

First of all, it was tested whether the base modifications in tRNA^Gly^ can be omitted without considerably compromising its affinity to EF-Tu. We radiolabeled and misacylated native (index n) tRNA^Gly^_n_ and unmodified (index u) tRNA^Gly^_u_ with five amino acids known to contribute only weakly to the overall binding energy of TCs (47) and checked TC formation in an EMSA (Figure 2A). TC formation was detected for both tRNAs acylated with the cognate glycine, the about equally contributing l-Leu and the stronger contributing l-Lys. Moreover, faint bandshifts were observed for native tRNA^Gly^_n_ misacylated with the weaker contributing l-Ala and l-Glu. Hence, detection of the TC with the cognate pair Gly-tRNA^Gly^_u_ marks the limit of detection in our assay system. Omission of base modifications in tRNA^Gly^ entails a small, but acceptable reduction of the affinity to EF-Tu.

To put the results of our assay into perspective with the published affinity contribution ranking of tRNA and l-amino acids (47), we performed EMSAs using five model tRNAs charged with glycine and 18 l-aa. These are unmodified tRNA^Gly^_u_ (fourth-strongest EF-Tu binder), native and unmodified tRNA^Tyr^ (tRNA^Tyr^_n_ and tRNA^Tyr^_u_, the weakest EF-Tu binder), and in addition two ‘affinity-transplanted’ tRNAs. The affinity of the tRNA moiety for EF-Tu is mainly determined by three base pairs of the TΨC stem, viz. 49–65, 50–64 and 51–63 (highlighted in Figure 1A). By transplantation of these base pairs from one tRNA to another, the acceptor tRNA obtains the properties of the donor tRNA with respect to EF-Tu binding strength (48–50). The affinity-transplanted tRNAs (index tp) are tRNA^Gly^_tp_ with the mentioned three base pairs transplanted from the weakest binder (tRNA^Tyr^) and tRNA^Tyr^_tp_ with the three base pairs taken from the third-strongest binder (tRNA^Asp^).

The results obtained with the high-affinity tRNA^Gly^_u_ (Figure 2B) show that the intensity of the bandshifts generally reflects the published affinity contribution ranking of l-amino acids (47). As expected, the transplanted tRNA^Gly^_tp_ shows a low affinity and TC formation was only detected with the three strongest contributing amino acids l-Gln, l-Trp and l-Tyr (Figure 2C). A similar pattern was observed with native tRNA^Tyr^_n_, here we found bandshifts only with the four strongest contributing l-aa (l-Gln, l-Trp, the cognate l-Tyr and l-Asn; Figure 2D) and no TC formation was observed with unmodified tRNA^Tyr^_u_ (Figure 2E). The transplanted tRNA^Tyr^_tp_ was expected to show a high affinity and we found that the binding pattern was rescued to the level of unmodified tRNA^Gly^_u_ or better (compare Figure 2F with B). The data are in good agreement with previous findings and in most cases, the limit of detection is close to the cognate pair (considering the donor tRNA in case of transplanted tRNAs). Cognate pairs are tuned to be optimal for translation; however, moderate reductions in affinity, which already are below the limit of detection of our assay, do not necessarily exert adverse effects on translation (49,51). Importantly, if any bandshift with d-aa-tRNA is detected, the binding energy must be at least close to the optimum.

Next, we acylated the high-affinity tRNAs tRNA^Gly^_u_ and tRNA^Tyr^_tp_ and the low affinity tRNAs tRNA^Gly^_tp_ and tRNA^Tyr^_u_ with 18 d-aa. Both high affinity tRNAs delivered detectable TCs with d-Ser, d-Phe and d-Pro, whereas no TCs were found for the low affinity tRNAs (Figure 2G and H, see also Supplementary Figure S1).

To facilitate tighter binding of d-aa-tRNA to EF-Tu, we introduced mutations into the EF-Tu amino acid binding pocket. Here, we replaced bulky for small amino acids of different character (such as Gly, Ala, Ser) to minimize anticipated steric hindrance of d-aa-tRNA binding (see Figure 1C). An earlier study had shown that translation with polycyclic l-amino acids, which apparently were too large to fit into the amino acid binding pocket of wild-type EF-Tu, was enabled by rational mutagenesis of the pocket (34). After initial tests with several mutants, we focused on the most promising mutants E215A, E215G, E215S, N273A and N273S. In summary, the mutants enabled unequivocal detection of TCs with d-Ala-, d-Ile- d-Pro- and d-Ser-tRNA^Gly^_u_, whereby each mutant bound to a different subset of these. Additionally, very faint bands of TCs were observed with d-Asn-, d-Gln-, d-His-, d-Phe- and d-Trp-tRNA^Gly^_u_ (Supplementary Figure S2). GFP synthesis in a defined, reconstituted translation system using either wild-type EF-Tu or the E215 and N273 mutants revealed a strongly reduced activity of all E215 mutants in translation. However, at least the EF-Tu mutant N273S could be considered as an acceptable candidate for incorporation of d-aa into proteins (Supplementary Figure S3).

So far, our results have demonstrated that even wild-type EF-Tu is generally able to form stable TCs with d-aa-tRNA, provided that the tRNA sufficiently compensates for the low contribution of the d-aa. This applies regardless of whether the tRNA shows an intrinsically strong affinity to EF-Tu or whether the high affinity is attained through engineering (‘transplantation’), which can be done to any tRNA. These results suggest to explore the incorporation of d-aa with unmodified tRNA^Gly^_u_.

### Incorporation of d-aa into peptides

To test d-amino acid incorporation into a polypeptide by the ribosome, we prepared a defined *in vitro* translation system analogous to the PURE system (36). The templates for our reporter peptides are derived from the N-terminal sequence of EF-Tu and encode the amino acid sequence fMSKAKFARTKPHANA[x]HHHHHH, whereby ‘x’ is the designated misincorporation site that consists of either one, two or three glycine codons in a row on the corresponding mRNA (templates designated ‘G_1_’, ‘G_2_’ and ‘G_3_’, respectively) or an opal stop codon (designated ‘O’). Since RRF together with elongation factor G (EF-G) and release factor 3 (RF3) can mediate premature peptidyl-tRNA drop-off if the ribosome pauses within the first six amino acids (52), we placed the misincorporation site ‘x’ at some distance to the N-terminus to ensure the nascent peptide is accommodated in the ribosome exit tunnel before d-aa incorporation ensues. Our system contains all individual components required for the translation of these peptides, with the exception of the amino acid glycine and the corresponding synthetase GlyRS. To decode the glycine codons, we added tRNA^Gly^_u_ misacylated with l- or d-aa; [^35^S]-Met was added for radiolabeling. The translation products were purified using Ni-NTA-agarose to eliminate non-incorporated ^35^S-Met, electrophoresed using tricine-SDS-PAGE and visualized by phosphorimaging.

In the main experiment, we assayed single, double and triple incorporation of 18 different d-aa using templates ‘G_1_’, ‘G_2_’ and ‘G_3_’, which we compared to the incorporation of the corresponding l-aa (glycine has no stereocenter, cysteine was omitted). Unexpectedly, double bands were detected on the gels and their origin was investigated (see Supplementary Figure S4). The lower band correlates with the 15-mer peptide fMSKAKFARTKPHANA that is terminated just before the misincorporation site and co-purifies most likely due to the single histidine at position 12. The upper band corresponds to the intended full-length product.

**Figure 3.**
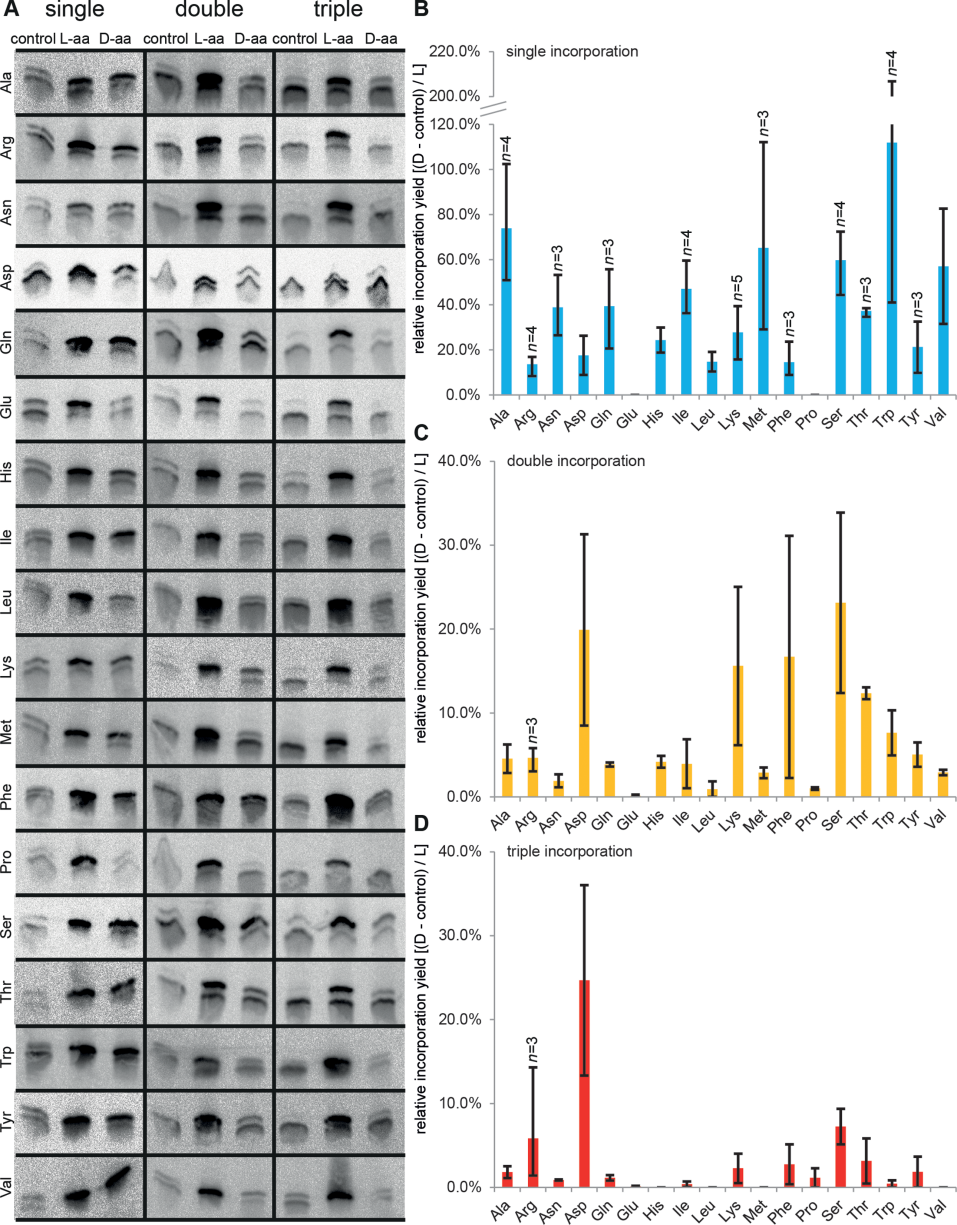
Incorporation of d-aa into peptides. (**A**) Tricine-SDS-PAGE analyses of single, double and triple incorporation of d-aa. Templates ‘G_1_’, ‘G_2_’ and ‘G_3_’ were translated in the presence of wild-type EF-Tu and tRNA^Gly^_u_ misacylated with the indicated d- or l-amino acid. (**B–D**) Relative incorporation efficiencies of d-aa and l-aa from single (B), double (C) and triple (D) incorporation experiments. The intensities of full-length product bands were assessed and background was subtracted globally. The control signal was subtracted from the d-aa signal and the resulting value divided by the l-aa signal. Data are shown as means of at least two independent experiments (*n* > 2 where indicated), error bars show the range of determined data.

Since a full-length product band may arise from unspecific readthrough of a hungry codon or due to small enantiomeric impurities found in the d-aa derivatives (l-aa content <0.1–0.96%), each reaction was accompanied by a control dedicated to show the maximum possible extent of unspecific full-length product formation. The control reactions contained the same absolute amounts of tRNA^Gly^_u_, precisely deacyl-tRNA^Gly^_u_ and 1% of the respective l-aa-tRNA^Gly^_u_ to simulate the worst-case enantiomeric impurity. For evaluation, we assessed the intensities of the full-length product bands and subtracted the control signals from the d-aa signals, which we then related to the l-aa signals (Figure 3). Despite the relatively high variance between replicates, the data clearly point into the same direction: d-amino acids can reproducibly be incorporated with a reasonable efficiency. The variance is related to the fact that each data point results from two independent aminoacylation reactions (charging of l- and d-amino acid) and three independent translation reactions (control, l-aa, d-aa), followed by purification and analysis. Replicates were in no case conducted in parallel.

**Figure 4.**
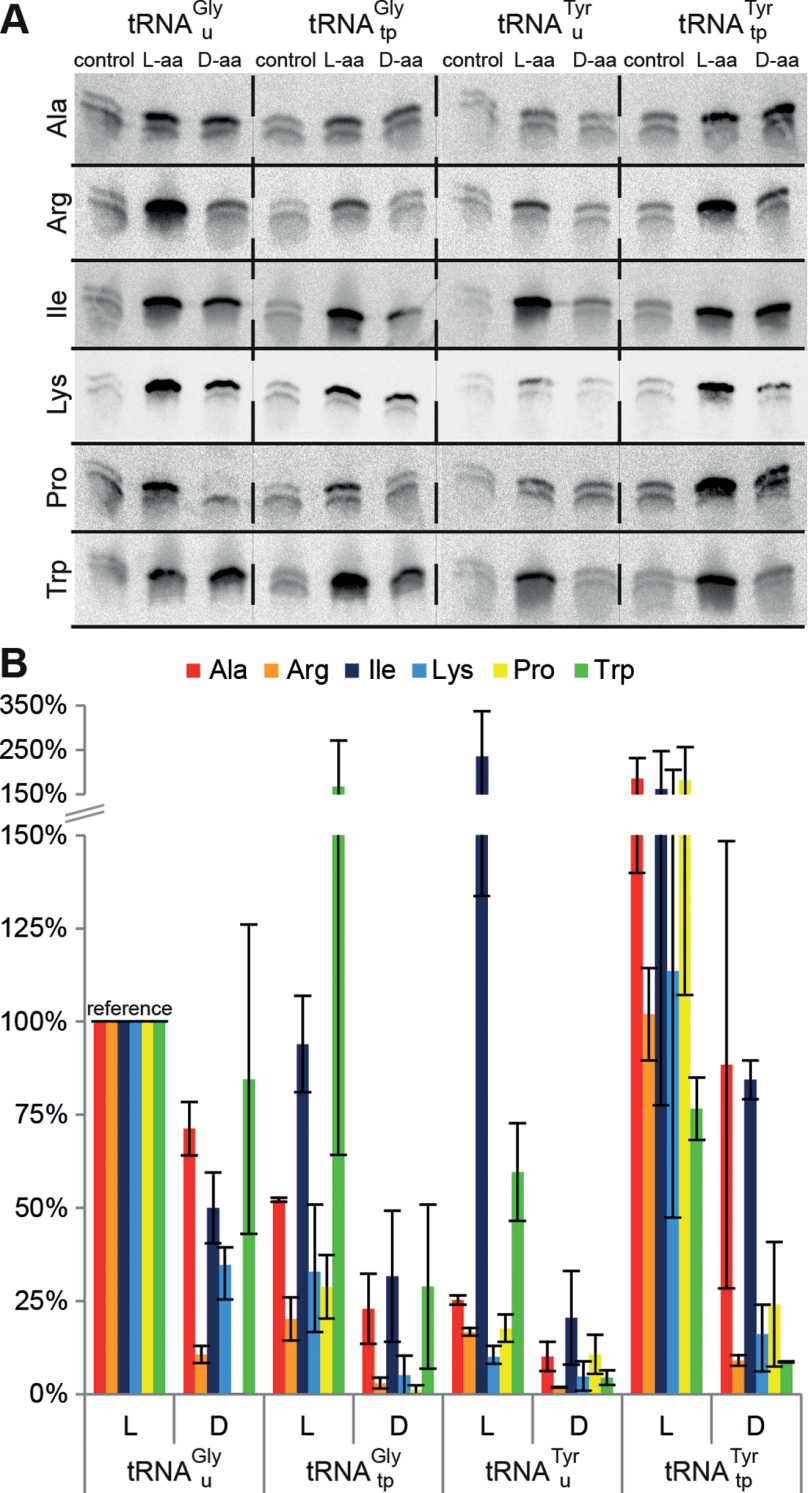
The effect of tRNA properties on d-aa incorporation. Single incorporation assays with four different tRNAs misacylated with six different l- and d-amino acids were performed in independent duplicates (triplicates for Lys). Template ‘G_1_’ was used for translation with tRNA^Gly^_u_ (high EF-Tu and A-site affinity) and tRNA^Gly^_tp_ (low EF-Tu affinity, high A-site affinity) and template ‘O’ for tRNA^Tyr^_u_ (low EF-Tu and A-site affinity) and tRNA^Tyr^_tp_ (high EF-Tu affinity, low A-site affinity). The anticodon on both tRNA^Tyr^ was changed to UCA to enable opal stop codon suppression. (**A**) Autoradiograms of the gels. Remarkable differences in the d-aa incorporation efficiencies depending on EF-Tu affinity become apparent. (**B**) Signal intensities normalized to the respective reaction with l-aa-tRNA^Gly^_u_ (lane 2 in (A)) are plotted. Control signals were subtracted from d-aa reaction signals prior to normalization. For clarity, the y-axis scaling is compressed above 150%. Error bars show the range of determined data.

In the single d-aa incorporation assays with tRNA^Gly^_u_, 16 of 18 tested d-aa could be incorporated, including five of the seven d-aa found to be resistant to translational elongation in an earlier study (28). No incorporation of d-Glu or d-Pro was observed; however, d-Pro could reproducibly be incorporated using tRNA^Tyr^_tp_ (see below). All 16 d-aa were incorporated with relative average efficiencies of at least 10%, twelve with ≥20%, nine with ≥30%, six with ≥40%, five with ≥50% and three with ≥60%.

In 14 cases, we observed incorporation of even two consecutive d-aa, although with a considerably reduced relative efficiency ranging between 3% and 23%, whereby d-Ser is most readily incorporated. The apparent high relative incorporation of d-Asp on the other hand does not reflect a strong incorporation of d-Asp but rather poor incorporation of l-Asp. Triple incorporations were also observed in some cases, but the absolute yield is clearly low. It was previously reported that the incorporation of two or more d-aa into a peptide strictly required that they are interspaced by at least two to three l-aa (28). Our results do not support such strict requirements; however, the incorporation of successive d-aa obviously poses an additional challenge over single incorporation.

d-aa incorporation via tRNA^Gly^_u_ apparently works, but this finding alone does not yet prove that its high affinity to EF-Tu and/or the ribosome is the key to permitting d-aa incorporation. To gain further insights, we performed single incorporation assays employing the previously characterized tRNAs tRNA^Gly^_u_, tRNA^Tyr^_tp_ (high EF-Tu affinity), tRNA^Gly^_tp_, and tRNA^Tyr^_u_ (low EF-Tu affinity) and templates ‘G_1_’ and ‘O’, respectively. The anticodons of both tRNA^Tyr^ were exchanged to UCA to enable opal stop codon suppression. As mentioned before, tRNA^Gly^ was described to show a high and amino acid independent affinity to the ribosomal A-site, whereas stable binding of tRNA^Tyr^ to the A-site requires a contribution of the esterified amino acid (46). We charged these tRNAs with six different d- and l-amino acids. Five of those d-aa (Arg, Ile, Lys, Pro, Trp) were described to be resistant to the ribosomal elongation event (28). Alanine, the smallest chiral amino acid, was used as a model for a d-aa which is largely unaffected by steric constraints in the ribosomal PTC (10). The results clearly show that a high affinity of the tRNA to EF-Tu is beneficial and in some cases mandatory for the incorporation of these d-aa (Figure 4). Likewise, the incorporation of l-aa contributing weakly to TC formation (i.e. Ala, Arg, Lys) and also l-Pro clearly benefits from a strongly contributing tRNA, whereas the low affinity tRNAs are well suited for strongly contributing l-Ile and l-Trp. No incorporation of d-Pro was observed with either tRNA^Gly^, yet a recognizable incorporation is seen with either tRNA^Tyr^; stronger with tRNA^Tyr^_tp_ than with tRNA^Tyr^_u_. This leaves d-Glu the only d-aa that could not be incorporated using our system. Interestingly, the incorporation of d-Trp via both tRNA^Tyr^ was poor, while it is easily achieved using either tRNA^Gly^. For this particular d-aa, ribosomal stereoselectivity rather than EF-Tu seems to be the decisive element. This might be related to the sheer size of the d-Trp side chain, provoking steric constraints in the PTC, and the low affinity of tRNA^Tyr^ to the A-site, which in combination may foster dissociation of the tRNA before the peptidyl transfer reaction can occur.

**Figure 5.**
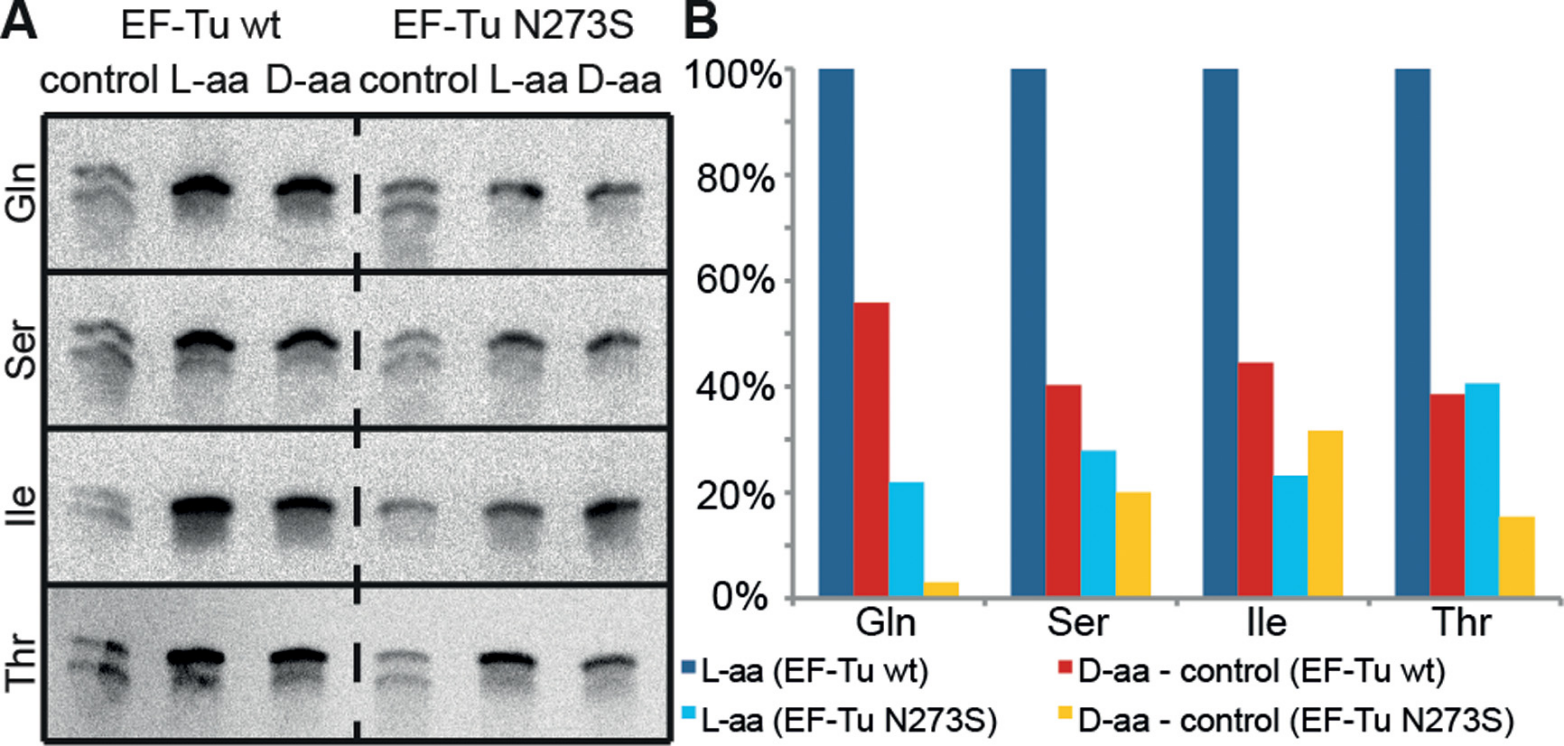
Tricine-SDS-PAGE analyses of single D-aa incorporation with wild-type EF-Tu and EF-Tu N273S. Template ‘G_1_’ was translated in the presence of tRNA^Gly^_u_ misacylated with selected d- and l-amino acids. (**A**) Autoradiograms of the gels. (**B**) Quantification of full-length product bands relative to bands from l-reactions with wild-type EF-Tu (lane 2 in (A)). EF-Tu N273S is inferior to the wild-type in all cases.

In preliminary experiments aimed at enhancing the yield of d-aa incorporation, we had screened several measures. Among these were the addition of EF-P (1 μM as used in (53)), which is known to resolve ribosome stalling at oligo-proline sequences (53,54); addition of EF4 (360 nM; 0.3 ribosome equivalents), which can help stalled ribosomes to resume translation by back-translocation especially when stalling is induced by an increased magnesium concentration (55–57); and raising the magnesium concentration from 13 to 18 mM to alter the ribosome's flexibility in the presence or absence of EF4. However, none of these measures showed a reproducible, beneficial effect (data not shown). We also included our most active EF-Tu mutant (N273S) into the test system and found that it was rather detrimental than beneficial for both the translation of all-l-peptides and mixed l-d peptides (Figure 5), leaving wild-type EF-Tu the best choice to incorporate d-amino acids. A likely reason is hyperstabilization of l-aa-tRNA by the EF-Tu mutant (compare Supplementary Figures S1 and S2), which is known to reduce the rate of peptide bond formation (49).

**Figure 6.**
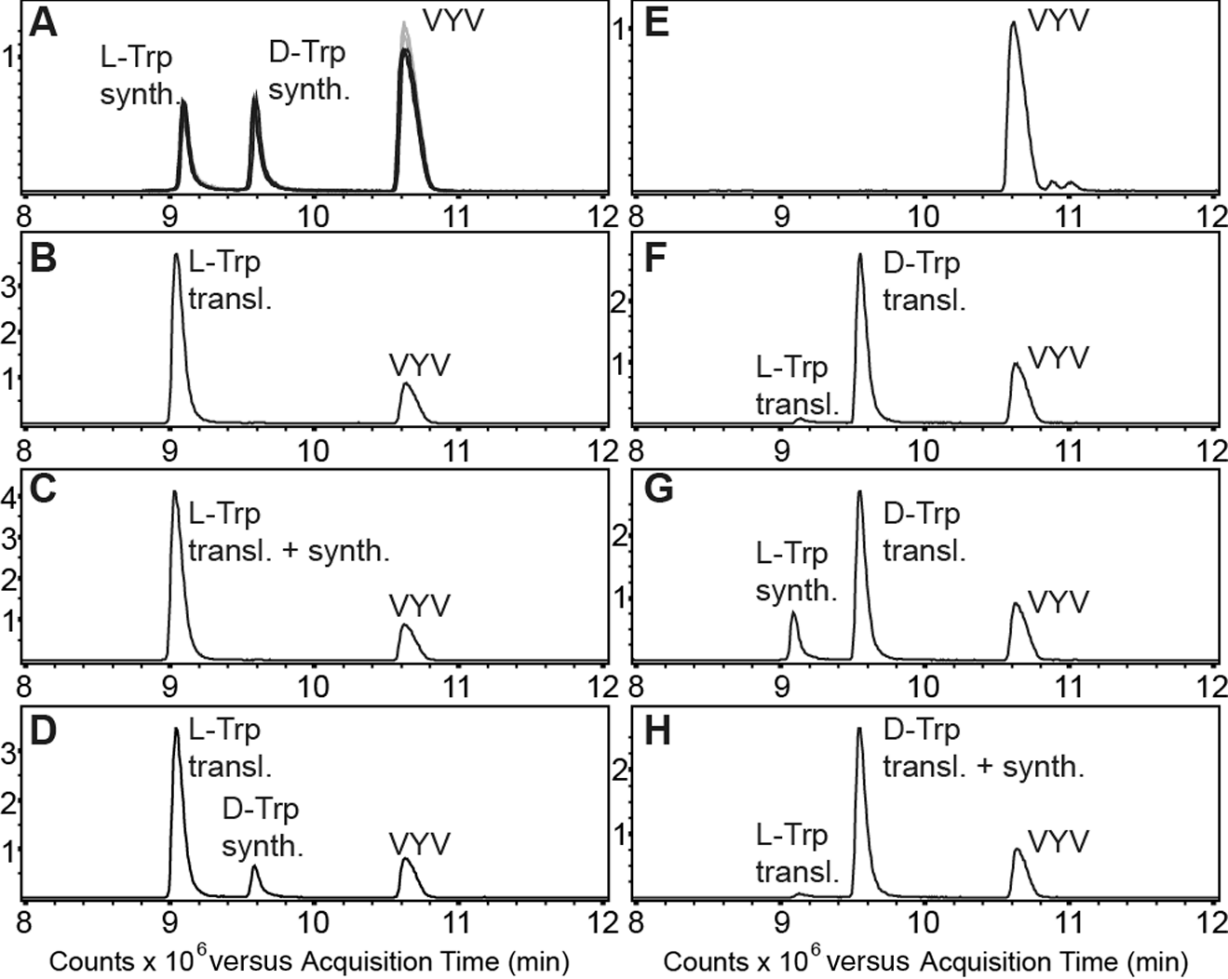
LC-MS analysis of translation products. The graphs show merged extracted ion chromatograms of the most intense charge states of the full-length peptides [(M+5H)^5+^: 539.6 *m/z*, (M+4H)^4+^: 674.3 *m/z*, (M+3H)^3+^: 898.7*m/z*; asymmetric peak detection (−0.3 Da/+0.7 Da); calculated monoisotopic mass (2693.3 Da) and of the synthetic tripeptide VYV (380.2 *m/z*). (**A**) Overlay of three standards each run before (grey) and after the samples (50 ng per peptide) (black). (**B**) Translated l-Trp peptide (∼350 ng) with VYV. (**C**) Translated l-Trp peptide with synthetic l-Trp peptide and VYV. The l-Trp peptides co-elute. (**D**) Translated l-Trp peptide with synthetic d-Trp peptide and VYV. The peptides are separated. (**E**) Negative control with VYV. (**F**) Translated d-Trp peptide (∼240 ng) with VYV. The d-Trp peptide elutes at the expected retention time, a minor peak corresponding to the l-Trp peptide (∼7 ng) is also detected. (**G**) Translated d-Trp peptide with synthetic l-Trp peptide and VYV. The peptides are separated. (**H**) Translated d-Trp peptide with synthetic d-Trp peptide and VYV. The d-Trp peptides co-elute.

Thus far, we have demonstrated several examples for the formation of full-length products in *in vitro* translation reactions in response to the addition of tRNA^Gly^_u_ charged with d-amino acids. To complement these results with additional direct evidence for d-aa incorporation, we made use of the fact that d-aa containing peptides and their all-l-counterparts are diastereomers, which generally show different retention times in reversed-phase HPLC analyses (58). We translated template ‘G_1_’ in the presence of deacyl-tRNA^Gly^_u_ (negative control), l-Trp-tRNA^Gly^_u_ or d-Trp-tRNA^Gly^_u_ and analyzed the translation products by LC-MS (Figure 6). Tryptophan was selected as an extreme model because ribosomal stereoselectivity has been described as a function of the size of the amino acid side chain (10) and tryptophan has the bulkiest one. Furthermore, the d-Trp derivative used for aminoacylation showed the highest contamination with the l-antipode (0.96%) among all d-aa derivatives used. The likelihood of false positives, which can be identified by this method, is therefore considered to be highest for this specific d-aa. The retention times of the expected products were determined using corresponding chemically synthesized peptides (Figure 6A). The tripeptide VYV was added to all samples as an internal standard and showed stable retention times through all measurements, so that we can exclude any retention time shifts due to column clogging. The translated peptides were detected at the expected retention times. Aliquots of the translation products were spiked with either of the synthetic peptides and co-elution or separation of synthetic and translated peptides was observed where expected, reassuring a correct assignment of the translation products. We observed strong single incorporation of d-Trp (≈70% of l-Trp incorporation calculated by peak integration) with only a minuscule amount of full-length product arising from the l-antipode contamination (Figure 6F). We also tested the incorporation of d-Ile and d-Val and identified the expected peptides, which showed the exact same mass but increased retention times compared to the corresponding peptides containing l-Ile and l-Val, respectively. In these cases, no product formation due to enantiomeric impurities of d-aa was detected at all (Supplementary Figure S5). These data strongly corroborate the results shown in Figures 3–5.

## DISCUSSION

Collectively, our results demonstrate a decent incorporation of almost all d-aa. Indirect evidence obtained by tricine-SDS-PAGE is accompanied by rigid controls dedicated to cancel out any unspecific full-length product formation due to unspecific readthrough, small enantiomeric impurities of the d-aa or misacylation of the tRNA used to transport d-aa by aaRS in the translation reaction. Direct evidence for d-aa incorporation obtained by LC-MS further substantiates the correctness of our results. Obviously, the ribosome is well able to form peptide bonds between l-aa and d-aa and vice versa, revealing an astonishing plasticity of the ribosomal PTC. Even the incorporation of consecutive d-aa appears to be feasible, although with a markedly reduced efficiency. Our *in vitro* translation system comprises exclusively native components from *E. coli*, i.e. aaRS, tRNAs, EF-Tu, ribosomes, and other translational factors, and thus keeps the overall accuracy for protein synthesis before and after the incorporation of the non-canonical amino acid (in our case d-aa). We have shown that the properties of the tRNA in terms of affinity to EF-Tu and the ribosomal A-site are major determinants for the outcome, which should generally be considered also for the incorporation of demanding non-canonical l-aa.

Previous studies aimed at ribosomal incorporation of d-aa used variants of tRNA^Phe^ (11,17,18) or tRNA^Asn^ (22,23,27,28) for the delivery of d-aa. tRNA^Phe^ shows an intermediate affinity to EF-Tu (47) and it is known that A-site binding is destabilized in the absence of base modifications and also in the absence of an esterified amino acid that contributes to the overall free binding energy (46). d-aa incorporation via tRNA^Phe^ has been demonstrated with mutant ribosomes (11), but not with wild-type ribosomes.

tRNA^Asn^ shows a weak affinity to EF-Tu (47). Many prokaryotes lack AsnRS, aminoacylation of tRNA^Asn^ is in these cases achieved by misacylation with l-Asp followed by transamidation of l-Asp-tRNA^Asn^ to l-Asn-tRNA^Asn^ (59). At least under *in vivo* conditions, the intermediate misacyl-tRNA l-Asp-tRNA^Asn^ is excluded from translation, because both tRNA^Asn^ and l-Asp (in contrast to l-Asn) contribute only weakly to the overall binding energy of the TC with EF-Tu•GTP (47,59). Therefore, tRNA^Asn^ does not appear to be well-suited for the delivery of weakly contributing amino acids such as d-aa. Using an amber-suppressor variant of tRNA^Asn^, Goto *et al*. showed that there is no incorporation of d-Cys or d-Met by translational elongation, but they obtained first signs for the incorporation of d-Ser (27), which to us appears to be the least demanding d-aa. After changing the codon of the misincorporation site from UAG to UCC and the anticodon in tRNA^Asn^ from CUA to GGA, the same group achieved single-incorporation of 12 different d-aa, including d-Cys and d-Met (28). In our interpretation, this switch of codon and anticodon strengthened the interaction within the codon:anticodon minihelix and thereby increased the affinity of tRNA^Asn^ to the A-site.

Unmodified tRNA^Gly^ has proven to be an excellent choice as a vehicle for d-aa: it is not only able to transport d-aa to the ribosome, probably in a TC with EF-Tu, but it also enables d-aa incorporation by the ribosomal PTC.

Our findings may serve as a starting point to further elucidate how stereoselectivity of the translational apparatus is established on a molecular level. In this regard, it would be exciting to see at near-atomic resolution, how a d-aa-tRNA is accommodated by EF-Tu and by the ribosome before and after peptide bond formation. Furthermore, we expect the techniques applied in this study can be useful in synthetic biology for at least two fields of applications:

- Significant advances have been achieved in the field of protein design, e.g. the *de novo, in silico* design of a functional transmembrane zinc–proton antiporter (60). We believe that some designers may find it useful to introduce d-aa at distinct positions. The designed protein could potentially be produced by *in vitro* translation.
- Established methods such as ribosome display (61,62), mRNA display (63) and related techniques sharing a defined *in vitro* translation system as a common core (64,65) are powerful tools for the *de novo* identification or affinity maturation of pharmaceutically interesting peptide ligands. The door to implement d-aa with these applications, which may give rise to new peptidic pharmaceuticals with improved properties, is open: one could use a reduced set of *in vitro* transcribed tRNAs instead of bulk tRNA to decode l-aa and thereby liberate several sense codons (Takuya Ueda, personal communication), which could be used for the decoding of d-aa. The d-aa could then be introduced via any orthogonal tRNA that meets the same criteria as tRNA^Gly^, namely tight EF-Tu binding, which can easily be achieved by adjusting the sequence of the TΨC-stem (49), and tight amino-acid independent A-site binding.

## SUPPLEMENTARY DATA

Supplementary Data are available at NAR Online.

SUPPLEMENTARY DATA
