# Supplementary material for: Outwitting EF-Tu and the ribosome: translation with d-amino acids
Source: Nucleic Acids Res. 2015 May 30;43(12):5687–98. doi: 10.1093/nar/gkv566 (PMC4499158; doi:10.1093/nar/gkv566)
Supplement: SUPPLEMENTARY DATA [file supp_43_12_5687__index.html]

Outwitting EF-Tu and the ribosome: translation with d-amino acids — Outwitting EF-Tu and the ribosome: translation with d-amino acids — SUPPLEMENTARY DATA 

# Outwitting EF-Tu and the ribosome: translation with d-amino acids

## SUPPLEMENTARY DATA

- SUPPLEMENTARY DATA
