# Supplementary material for: Outwitting EF-Tu and the ribosome: translation with d-amino acids
Source: Nucleic Acids Res. 2015 May 30;43(12):5687–98. doi: 10.1093/nar/gkv566 (PMC4499158; doi:10.1093/nar/gkv566)
Supplement: SUPPLEMENTARY DATA [file supp_gkv566_nar-00918-h-2015-File008.pdf]

Supplementary Data

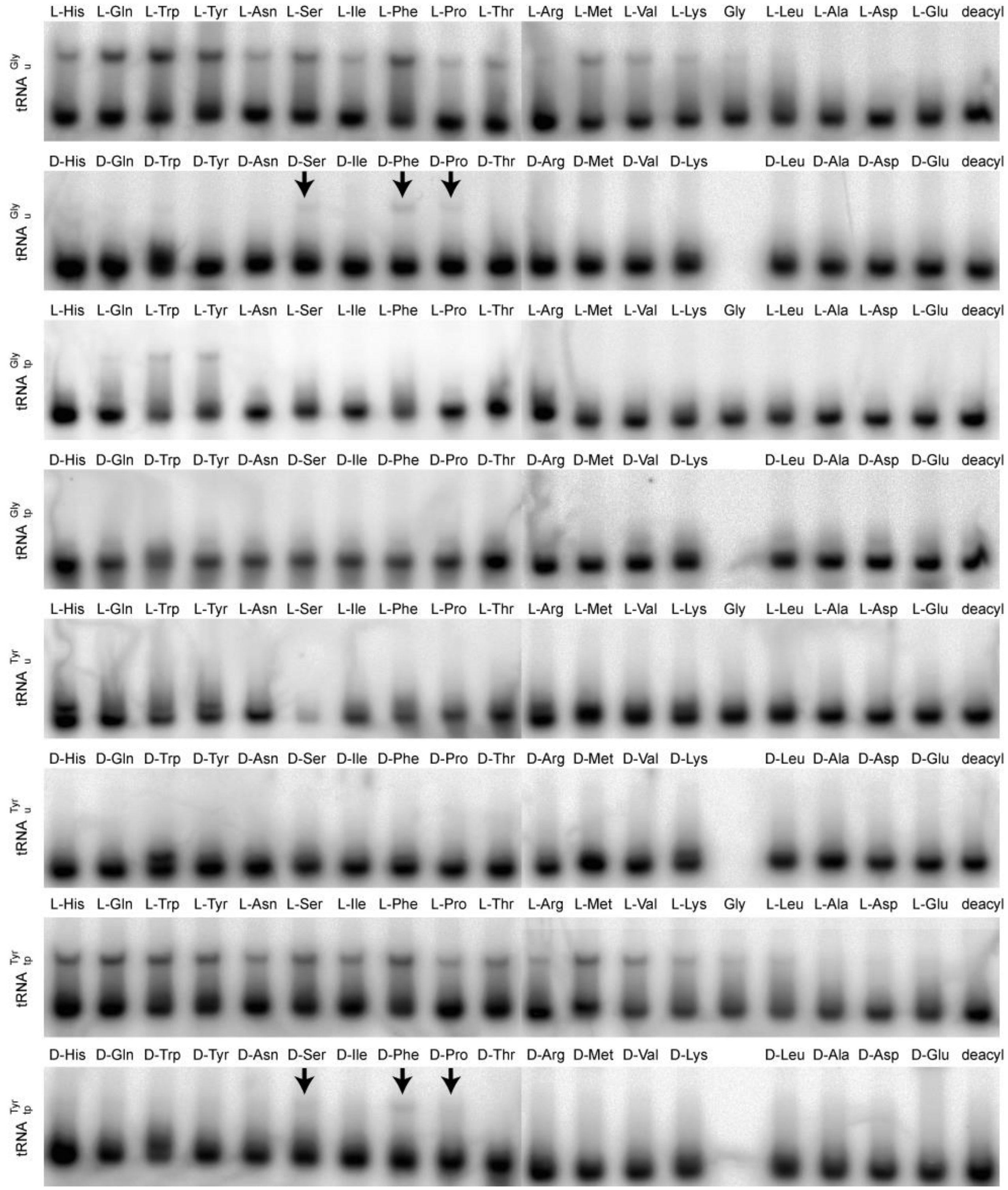

Figure S1. EMSAs with wild-type EF-Tu and four tRNAs misacylated with L-and D-amino acids. Arrows highlight ternary complex detection with D-aa-tRNA. For evaluation, see **Fig. 2**.

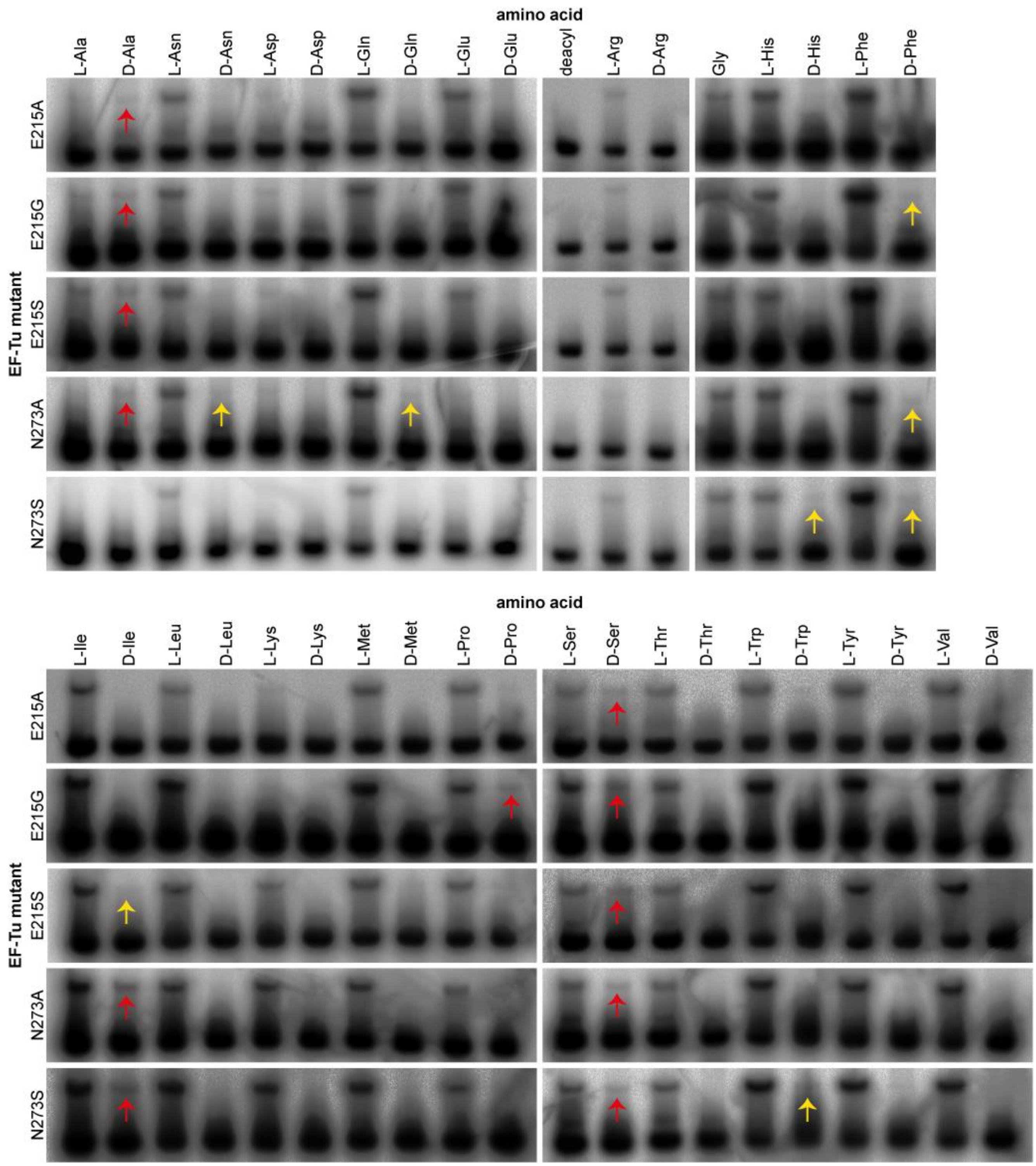

Figure S2. EMSAs with mutant EF-Tu and L-aa- or D-aa-tRNA$^{Gly}_{u}$. Red and yellow arrows highlight clearly visible and barely visible ternary complexes with D-aa-tRNA, respectively.

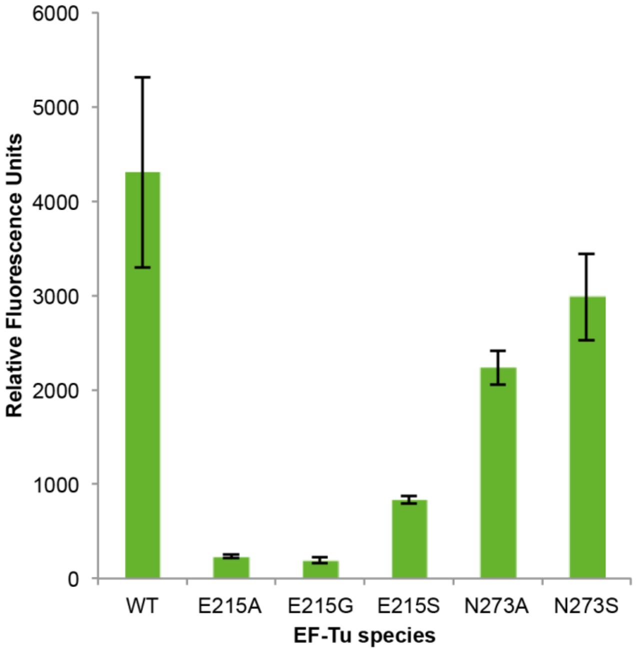

Figure S3. GFP Translation with wildtype and mutant EF-Tu. Emerald GFP was translated in the presence of the indicated EF-Tu species in independent duplicates. The mean endpoint fluorescence intensity is plotted, error bars show the range of determined data.

In the main text, we have assigned the lower band the 15-mer peptide fMSKAKFARTKPHANA and the upper band the full-length product fMSKAKFARTKPHANAxHHHHHH. This is corroborated by the following evidence: If we translate template 'O' in presence of RF2 to produce a defined stop after 15 amino acids, we find only the lower band (**Figure S4A** and **B,** lane 5), while we find exclusively the upper band if we translate template 'G_1' in presence of glycine and GlyRS (lane 4). Translating template 'G_1' in absence of glycine/GlyRS without or with added deacyl-$tRNA^{Gly}_u$ yields the lower band and a weak upper band (lanes 1 and 2, respectively). The weak upper band observed under these conditions most likely arises from unspecific readthrough of the hungry codon at position 16 during the prolonged incubation time (3 hours at 37°C); we observed the same effect with a hungry tyrosine-specific codon at position 16 and with template 'O' in absence of RF2 (not shown). If we additionally omit L-histidine, we find no band at all (lane 3), suggesting that the histidine residue at position 12 is essential for co-purification of the lower band. The LC-MS data shown in Figure 6 demonstrated the formation of full-length products in response to the addition of L-Trp-$tRNA^{Gly}$ or D-Trp-$tRNA^{Gly}$ to the translation reaction. In the negative control reaction of that experiment, which contained only deacyl-$tRNA^{Gly}$, we were able to detect an abundant mass that precisely matches the calculated mass of the proposed 15-mer peptide (m_{calc} = 1684.88 Da) and heavier isotopes of it (**Figure S4C**).

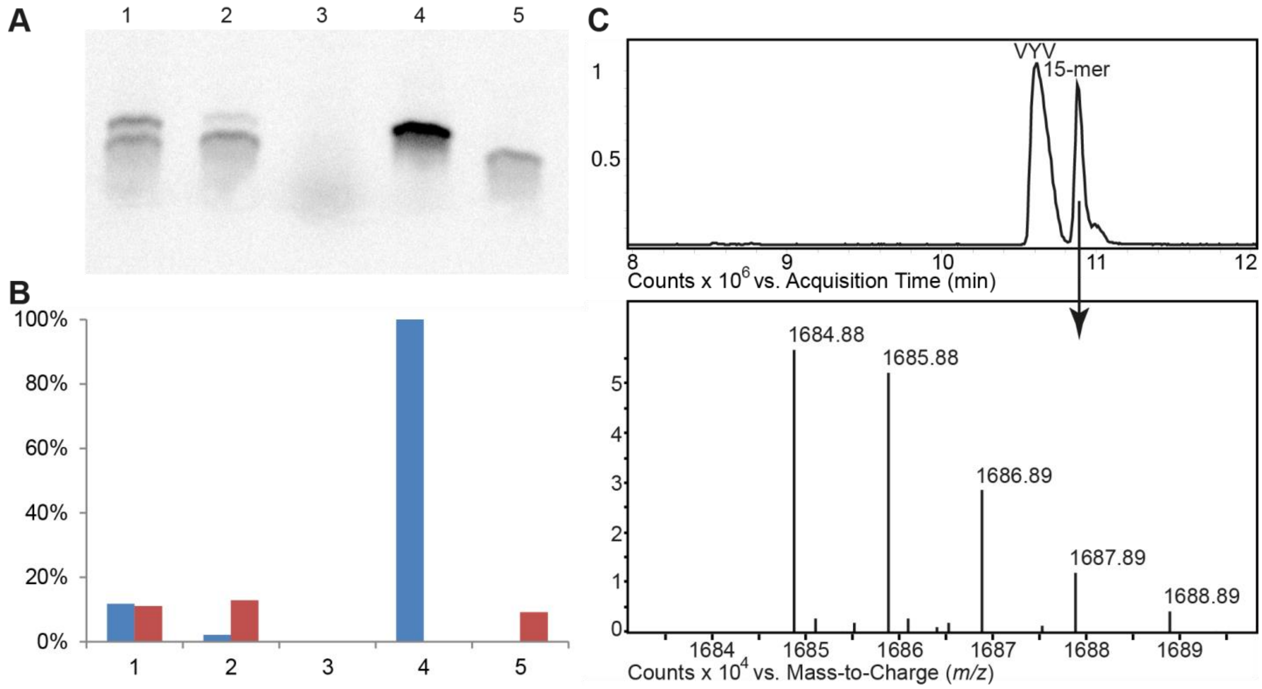

Figure S4. Assignment of bands observed on gels of ^{35}S-Met labelled translation products. (A) template 'G_1' was translated in absence of glycine & GlyRS (lane 1), in absence of glycine & GlyRS with added deacyl-$tRNA^{Gly}_u$ (lane 2), in absence of glycine, GlyRS and L-histidine (lane 3) and in presence of both glycine and GlyRS (lane 4). Template 'O' was translated in presence of RF2 (lane 4). (B) relative quantitation of the upper bands (blue bars) lower bands (red bars) versus the upper band in lane 4. (C) upper panel: merged extracted ion chromatogram from an LC-MS analysis of a translation reaction corresponding to lane 1 in panel a, showing the most intense charge states of the 15-mer peptide fMSKAKFARTKPHANA (M = 1684.88 Da; $(M+3H)^{3+}$ ion = 562.6 *m/z* shown) and the synthetic tripeptide VYV (380.2 *m/z*), which was added as an internal standard. Lower panel: deconvolution of the peak at 10.8 minutes shows the expected mass of the 15-mer peptide and isotopes thereof.

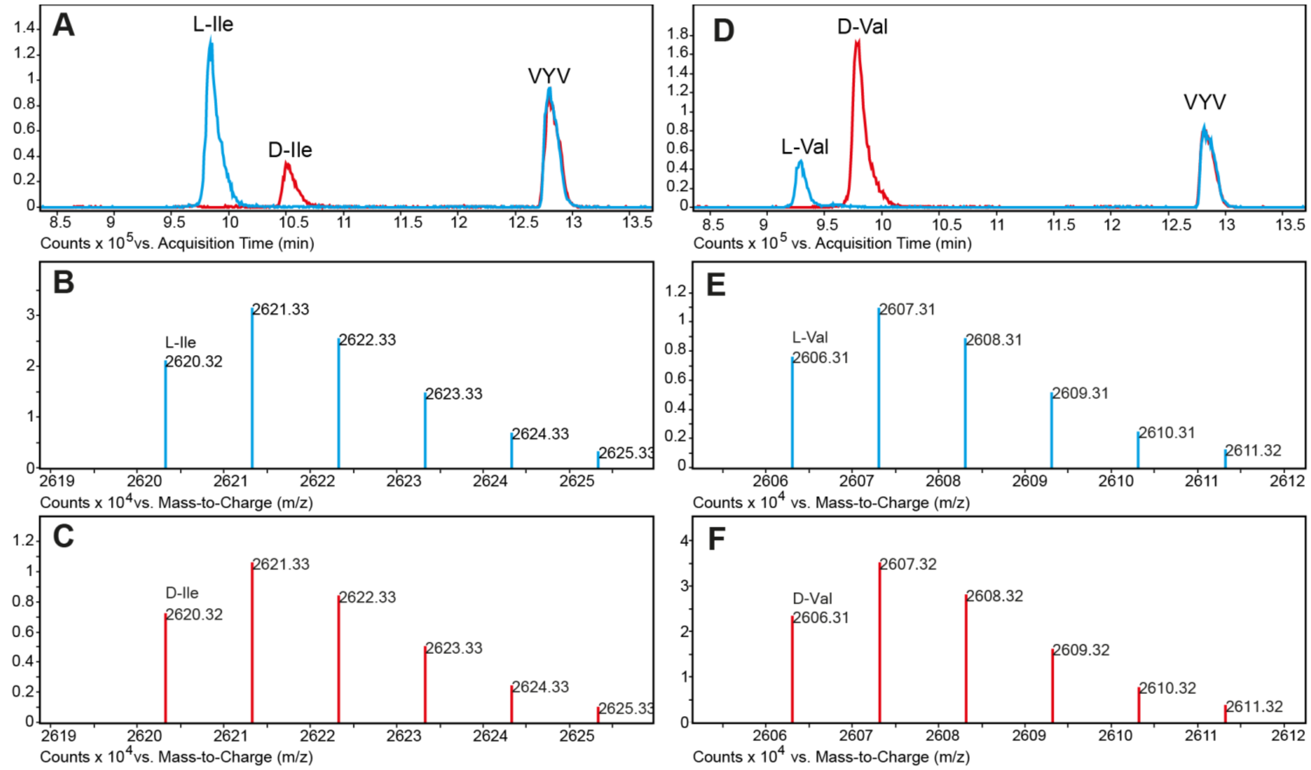

Figure S5. LC-MS analysis of translation products. Template G_1 was translated in presence of $tRNA^{Gly}{}_u$ misacylated with either L-Ile, D-Ile, L-Val or D-Val. The purified translation products were spiked with the tripeptide VYV and analysed by LC-MS. **A**, overlay of merged extracted ion chromatograms (EICs) of the most intense charge states of the full length peptides containing L- or D-Ile [$(M+5H)^{5+}$: 525.06 *m/z*, $(M+4H)^{4+}$: 656.08 *m/z*, $(M+3H)^{3+}$: 874.4*m/z*; symmetric peak detection ±10 ppm; calculated monoisotopic mass 2620.32 Da) and of the synthetic tripeptide VYV (380.2 *m/z*). The L-Ile peptide is detected at a retention time of 9.8 minutes (cyan line), the D-Ile peptide is detected at 10.6 minutes (red line). The L-Ile peptide is not detected in the D-Ile sample and *vice versa*. The VYV peptide shows stable retention times in both samples. **B**, deconvolution spectrum of the L-Ile peptide. **C**, deconvolution spectrum of the D-Ile peptide. Both deconvolution spectra show the exact calculated monoisotopic mass of the expected peptides and heavier isotopes thereof. **D**, overlay of merged extracted ion chromatograms (EICs) of the most intense charge states of the full length peptides containing L- or D-Val [$(M+5H)^{5+}$: 522.26 *m/z*, $(M+4H)^{4+}$: 652.57 *m/z*, $(M+3H)^{3+}$: 869.77 *m/z*; symmetric peak detection ±10 ppm; calculated monoisotopic mass 2606.3 Da) and of the synthetic tripeptide VYV (380.2 *m/z*). The L-Val peptide is detected at a retention time of 9.3 minutes (cyan line), the D-Ile peptide is detected at 9.8 minutes (red line). The L-Val peptide is not detected in the D-Val sample and vice versa. The VYV peptide shows stable retention times in both samples. **E**, deconvolution spectrum of the L-Ile peptide. **F**, deconvolution spectrum of the D-Ile peptide. Both deconvolution spectra show the exact calculated monoisotopic mass of the expected peptides and heavier isotopes thereof.

For comparison: the peak height of 50 ng L-Trp- or D-Trp-Peptide in the EIC with the symmetric peak detection settings applied here is 1×10^4.

Supplementary Methods

Translation templates

Templates 'G1', 'G2', 'G3' and 'O' were produced by gene synthesis and subcloning into the vector **pIDT-SMART** (IDT). The following sequences were synthesized (coding sequence underlined, designated misincorporation site highlighted in red):

'G$_1$': TAATACGACTCACTATAGGGTCTAGAAATAATTTTGTTTAACTTTAAGAAGGAGATATACCAATGTCGAAA
GCCAAATTCGCCCGGACCAAACCGCACGCCAACGCC**GGG**CACCACCACCACCACCACTAATGAGGATCCCGGGA
ATTCTCGAGTAAGGTTAACCTGCAGGAGGCCTTTAATTAAGGTGGTGCGGCCGCGCTAGCGGTCCCGGGGGATC
GATCCGGCTGCTAACAAAGCCCGAAAGGAAGCTGAGTTGGCTGCTGCCACCGCTGAGCAATAACTAGCATAACC
CCTTGGGGCCTCTAAACGGGTCTTGAGGGGTTTTTTGCTGAAAGGAGGAACTATAT

'G$_2$': TAATACGACTCACTATAGGGTCTAGAAATAATTTTGTTTAACTTTAAGAAGGAGATATACCAATGTCGAAA
GCCAAATTCGCCCGGACCAAACCGCACGCCAACGCC**GGGGGG**CACCACCACCACCACCACTAATGAGGATCCCG
GGAATTCTCGAGTAAGGTTAACCTGCAGGAGGCCTTTAATTAAGGTGGTGCGGCCGCGCTAGCGGTCCCGGGGG
ATCGATCCGGCTGCTAACAAAGCCCGAAAGGAAGCTGAGTTGGCTGCTGCCACCGCTGAGCAATAACTAGCATA
ACCCCTTGGGGCCTCTAAACGGGTCTTGAGGGGTTTTTTGCTGAAAGGAGGAACTATAT

'G$_3$': TAATACGACTCACTATAGGGTCTAGAAATAATTTTGTTTAACTTTAAGAAGGAGATATACCAATGTCGAAA
GCCAAATTCGCCCGGACCAAACCGCACGCCAACGCC**GGGGGGGGG**CACCACCACCACCACCACTAATGAGGATC
CCGGGAATTCTCGAGTAAGGTTAACCTGCAGGAGGCCTTTAATTAAGGTGGTGCGGCCGCGCTAGCGGTCCCGG
GGGATCGATCCGGCTGCTAACAAAGCCCGAAAGGAAGCTGAGTTGGCTGCTGCCACCGCTGAGCAATAACTAGC
ATAACCCCTTGGGGCCTCTAAACGGGTCTTGAGGGGTTTTTTGCTGAAAGGAGGAACTATAT

'O': TAATACGACTCACTATAGGGTCTAGAAATAATTTTGTTTAACTTTAAGAAGGAGATATACCAATGTCGAAA
GCCAAATTCGCCCGGACCAAACCGCACGCCAACGCC**TGA**CACCACCACCACCACCACTAATGAGGATCCCGGGA
ATTCTCGAGTAAGGTTAACCTGCAGGAGGCCTTTAATTAAGGTGGTGCGGCCGCGCTAGCGGTCCCGGGGGATC
GATCCGGCTGCTAACAAAGCCCGAAAGGAAGCTGAGTTGGCTGCTGCCACCGCTGAGCAATAACTAGCATAACC
CCTTGGGGCCTCTAAACGGGTCTTGAGGGGTTTTTTGCTGAAAGGAGGAACTATAT

Cloning, Expression and Purification of Recombinant Proteins.

Genes of translation factors were amplified from E. coli K12 MG1655 genomic DNA (GenBank U00096.2) using the Expand HiFi PCR system (Roche) and cloned and fused to affinity tags (Strep-Tag II or TwinStrep-Tag) using the StarGate system (IBA GmbH), whereby all start codons were set to ATG. Primer sequences are given below. The following vectors (IBA GmbH) were used for the expression of recombinant proteins:

pASG-IBA5 (anhydrotetracycline-inducible, N-terminal StrepTag) for initiation factor 1 (IF1), initiation factor 2 (IF2), initiation factor 3 (IF3).

pASG-IBA105 (anhydrotetracycline-inducible, N-terminal Twin-Strep Tag) for alanyl-tRNA synthetase (AlaRS), arginyl-tRNA synthetase (ArgRS), asparaginyl-tRNA synthetase (AsnRS), glycyl-tRNA synthetase (GlyRS)*, isoleucyl-tRNA synthetase (IleRS), phenylalanyl-tRNA synthetase (PheRS)*, threonyl-tRNA synthetase (ThrRS), inorganic pyrophosphatase (iPPase), release factor 2 (RF2)

pASG-IBA3 (anhydrotetrycycline-inducible, C-terminal StrepTag) for elongation factor G (EF-G), elongation factor Ts (EF-Ts), release factor 1 (RF1), ribosome recycling factor (RRF), methionyl-tRNA synthetase (MetRS), methionyl-tRNA formyltransferase (MTF), tRNA nucleotidyltransferase (TRNT), adenosine diphosphate kinase (ADK), nucleoside diphosphate kinase (NDK),

pASG-IBA103 (anhydrotetracycline-inducible, C-terminal Twin-Strep Tag) for aspartyl-tRNA synthetase (AspRS), cysteinyl-tRNA synthetase (CysRS), glutaminyl-tRNA synthetase (GlnRS), glutamyl-tRNA synthetase (GluRS), histidinyl-tRNA synthetase (HisRS), leucyl-tRNA synthetase (LeuRS), lysyl-tRNA synthetase (LysRS), prolyl-tRNA synthetase (ProRS), seryl-tRNA synthetase (SerRS), tryptophanyl-tRNA synthetase (TrpRS), tyrosyl-tRNA synthetase (TyrRS), valyl-tRNA synthetase (ValRS), elongation factor 4 (EF4), elongation factor Tu (EF-Tu) and mutants thereof, release factor 3 (RF3).

* GlyRS consists of two only loosely coupled chains, tagging only one entails loss of the other during affinity chromatography. Thus, both encoding cistrons were first cloned into **pASG-IBA103** to fuse the β-subunit to a C-terminal TwinStrep-tag and the fused gene was subcloned into **pASG-IBA105** to fuse the α-subunit to an N-terminal TwinStrep-tag. PheRS also consists of two subunits that are tightly coupled, so one tag is sufficient.

The gene *lepA* encoding EF4 was synthesized and subcloned into a StarGate-compatible vector by GeneArt. The gene encoding RF2 harbors an inframe stop codon, expression of the protein requires a frameshift. This necessity was eliminated by site directed mutagenesis. All mutageneses were performed using the QuikChange Lightning site directed mutagenesis kit (Agilent). For verification, all plasmids were sequenced (LGC Genomics).

Expression plasmids were transformed into *E.coli* NEB Express (New England Biolabs). Cells were grown for ≈20 h at 30°C in 50-200 ml of EnPresso medium (BioSilta), expression was then induced for 24 h by the addition of anhydrotetracycline to 200 ng/ml, the temperature was increased to 37°C. For RF3 expression, cells were grown to ≈0.7 OD_{600}/ml at 37°C in 2YT-medium and induced for 4 h.

Cells were harvested by centrifugation (20 min, 4°C, 6,000 x g), resuspended in Buffer W (100 mM Tris-HCl pH 8.0, 150 mM NaCl, 1 mM EDTA) and lysed by two or three passages at ≈500 bar through a French®Press (Thermo Electron). Cell debris was pelleted by centrifugation (30 min, 4°C, 15,500 x g). The supernatant was filtered through 0.8/0.2 µm syringe filters (PALL) and subjected to affinity chromatography (AC) over StrepTrap HP 5 ml columns using an ÄKTA Express instrument (GE Healthcare). Buffer W was used for binding and washing, elution was done in the same buffer supplemented with 2.5 mM desthiobiotin (IBA GmbH). For all except IF1 and IF3, fractions were pooled and diluted in 20 mM Tris-HCl pH 8.0, 10 mM KCl and subjected to anion exchange chromatography over HiTrap Q HP 5 ml columns (GE healthcare) in the same buffer with a gradient from 10 mM to 500 mM KCl during 20 column volumes (CV). Purification of both EF-Tu and EF-Ts required a 40 CV gradient, as these proteins co-elute from AC and have a near-identical pI.

IF2 co-elutes from AC with 30S ribosomal subunits. 16S rRNA was eliminated by anion exchange. Ribosomal proteins were subsequently eliminated by a second AC.

AC-fractions of IF1 and IF3 were diluted in 50 mM HEPES-KOH pH 6.8, 10 mM KCl and purified by cation exchange over HiTrap SP 5 ml columns (GE Healthcare) with a gradient from 10 mM to 500 mM NaCl over 20 CV in the same buffer. IF1 was further purified by gel filtration chromatography in HEPES-KOH pH 6.8, 10 mM NCl over a HiLoad 16/60 SUPERDEX 75 column (GE Healthcare) and another round of cation exchange.

Purity of the proteins was determined by 10% Bis-Tris SDS-PAGE (novex). Each fraction was controlled for RNase-activity using the RNaseAlert QC system (Ambion). Impure fractions were either re-purified or discarded. Pure fractions were pooled and concentrated using Vivaspin 20 concentrator columns (Sartorius Stedim) with appropriate molecular weight cutoff. Before publication of ref (1), concentrates were supplemented with 1 volume of glycerol and stored at -20°C; except for IF1, all proteins were stable and active for years under these conditions. Since then, concentrates were buffer-exchanged into stock buffer (50

mM HEPES-KOH pH 7.6 @ 0°C, 100 mM KCl, 10 mM $MgCl_2$, 7 mM β-mercaptoethanol, 30% glycerol) by buffer addition and re-concentration, and were finally flash-frozen in liquid nitrogen and stored in aliquots at -80°C.

To determine specific protein concentrations, dilutions of the proteins and serial dilutions of bovine serumalbumine (Pierce) were subjected to 10% Bis-Tris SDS-PAGE (novex). Gels were stained with SyproRed (Molecular Probes) according to the manufacturer's instructions and scanned using a MolecularImager FX (Bio-Rad). Band intensities were quantified using ImageLab software (Bio-Rad). This method is independent of the amino acid composition of the proteins, inert to any components of the protein storage buffer and does not co-quantify contaminating proteins of different size if any are present.

Assembly of the translation system

Solution 1 (10x) contains 500 mM HEPES, 1 M potassium acetate, [20 mM ATP, 20 mM GTP, 10 mM CTP, 10 mM UTP (Jena Bioscience)], 200 mM creatine phosphate (Sigma), 1 mM 10-formyl-5,6,7,8 tetrahydrofolate (fTHF), 15 mg/ml bulk tRNA from *E. coli* MRE600 (Roche), [20 mM spermidine, 10 mM dithiothreitol (Applichem)]. fTHF was prepared from folinic acid calcium salt (Applichem) as described (1). Prior to addition, bulk tRNA was deacylated following ref (2) by incubation at 37°C for one hour in 0.05 mM sodium borate and desalted in H_2O over a NAP5 column (GE Healthcare). Before the addition of spermidine and DTT, the pH was adjusted to 7.60 on ice using KOH. Meeting the final volume required concentration in a Speedvac in between.

Solution 2 (16.67x) contains 5 mM each of the amino acids required for the intended purpose. The pH was adjusted to 7.60 on ice using acetic acid or KOH (depending on the composition).

Solution 3 (20x) contains 20.4 µM IF1, 8 µM IF2, 9 µM IF3, 12.6 µM EF-G, 31.6 µM EF-Ts, 4.8 µM RF-1, 3.2 µM RF-3, 9.4 µM RRF, 80 µg/mL creatine kinase (Roche), 2.4 µM ADK, 1.3 µM NDK, 280 µM iPPase, 100 µg/ml T7 RNAP (Stratagene), and 24 µM reassociated 70S ribosomes. For assembly, proteins were mixed in an Amicon Ultra 0.5 ml centrifugal filter column (3 kDa MWCO, Millipore) and concentrated. Stock buffer (see above) was added to 450 µl and concentrated again. Ribosomes were added last to make sure that they will not dissociate due to low magnesium conditions. Several cycles of concentration and addition of stock buffer were performed, until a buffer exchange of at least 99% was achieved. The desired final volume was adjusted with stock buffer. Single-use aliquots were flash-frozen in liquid nitrogen and stored at -80°C.

Solution 4 (20X) contains the aminoacyl-tRNA synthetases required for the intended purpose. A complete solution 4 comprises 14.6 µM <u>AlaRS</u>, 0.6 µM <u>ArgRS</u>, 13.6 µM <u>AsnRS</u>, 2.6 µM AspRS, 0.4 µM CysRS, 1.2 µM <u>GlnRS</u>, 4.6 µM GluRS, 1.8 µM GlyRS, 0.4 µM <u>HisRS</u>, 8 µM IleRS, 0.8 µM LeuRS, 2.2 µM <u>LysRS</u>, 0.6 µM <u>MetRS</u>, 13.6 µM <u>PheRS</u>, 3.2 µM <u>ProRS</u>, 0.8 µM <u>SerRS</u>, 1.8 µM <u>ThrRS</u>, 0.6 µM TrpRS, 0.4 µM TyrRS and 0.4 µM ValRS. Assembly and storage was done as described for solution 3 above. The aaRS present in translation experiments of templates G_1, G_2, G_3 and O are underlined.

Oligonucleotide synthesis

All primers, Flexizymes and unmodified tRNAs were synthesized by solid phase oligonucleotide synthesis using standard phosphoamidite chemistry on an ABI 392 (Applied Biosystems) or ÄKTA10 oligonucleotide synthesizers (GE-Healthcare). For RNA synthesis 2'-TBDMS protected phosphoamidites were used (3-5). Phosphoamidites and CPGs were purchased from Proligo and Thermo Fisher and used according to the manufacturer's instruction. Ultrafast-, rT-, pseudo-rU-, 4-thio-rU-, dihydro-rU- as well as bis-cyanoethyl-amidites were from ChemGenes. Identity and purity of all oligonucleotides was confirmed by RP- and IEX-HPLC and LC-MS (ESI-).

Primer sequences

Gene	Protein	Primer	
cca	TRNT	forward	pAATGAAGATTTATCTGGTCGGTGGTGCTGTTCGGG
		reverse	pTCCCTTCAGGCTTTGGGCAACGTTGTT
infA	IF1	forward	pAATGGCCAAAGAAGACAATATTGAAAT
		reverse	pTCCCGCGACTACGGAAGACAATGC
infB	IF2	forward	pAATGACAGATGTAACGATTAAAACGC
		reverse	pTCCCAGCAATGGTACGTTGGATCTC
infC	IF3	forward	pAATGAAAGGCGGAAAACGAGTTCAA
		reverse	pTCCCCTGTTTCTTCTTAGGAGCGAG
fusA	EF-G	forward	pAATGGCTCGTACAACACCCATCG
		reverse	pTCCCTTTACCACGGGCTTCAATTAC
tufB	EF-Tu	forward	pAATGTCTAAAGAAAAGTTTGAACGTAC
		reverse	pTCCCGCTCAGAACTTTTGCTACAAC
	EF-Tu E215A	QuikChange 1	TTCCTGCTGCCGATCGCAGACGTATTCTCCATC
		QuikChange 2	GATGGAGAATACGTCTGCGATCGGCAGCAGGAA
	EF-Tu E215G	QuikChange 1	CCTGCTGCCGATCGGCGACGTATTCTCCATC
		QuikChange 2	GATGGAGAATACGTCGCCGATCGGCAGCAGG
	EF-Tu E215S	QuikChange 1	CGTTCCTGCTGCCGATCAGCGACGTATTCTCCATCTC
		QuikChange 2	GAGATGGAGAATACGTCGCTGATCGGCAGCAGGAACG
	EF-Tu N273A	QuikChange 1	GGCCGTGCTGGTGAGGCCGTAGGTGTTCTGCT
		QuikChange 2	AGCAGAACACCTACGGCCTCACCAGCACGGCC
	EF-Tu N273S	QuikChange 1	GAAGGCCGTGCTGGTGAGAGCGTAGGTGTT
		QuikChange 2	AACACCTACGCTCTCACCAGCACGGCCTTC
tsf	EF-Ts	forward	pAATGGCTGAAATTACCGCATCCCT
		reverse	pTCCCAGACTGCTTGGACATCGCAG
prfA	RF1	forward	pAATGAAGCCTTCTATCGTTGCCAAACTGGA
		reverse	pTCCCTTCCTGCTCGGACAACGCCG
prfB	RF2	forward	pAATGTTTGAAATTAATCCGGTAAATAATCGC
		reverse	pTCCCTCATAACCCTGCTTTCAAACTTGCTTCGATAAATT
		QuikChange 1	CGACGTTCTTAGGGGGTATCTTGACTACGACGCC
		QuikChange 2	GGCGTCGTAGTCAAGATACCCCCTAAGAACGTCG
prfC	RF3	forward	pAATGACGTTGTCTCCTTATTTGCAAG
		reverse	pTCCCATGCTCGCGGGTCTGGTG
frr	RRF	forward	pAATGATTAGCGATATCAGAAAAGATGC
		reverse	pTCCCGAACTGCATCAGTTCTGCTTC
adk	ADK	forward	pAATGCGTATCATTCTGCTTGGCGCTCCGGGCGCGGG
		reverse	pTCCCGCCGAGGATTTTTTCCAGATCAGCGCGAACTTC
ndk	NDK	forward	pAATGGCTATTGAACGTACTTTTTCCATCATCAAAC
		reverse	pTCCCACGGGTGCGCGGGCACACTTCGCCTTCGCCAA
ppa	PPA	forward	pAATGAGCTTACTCAACGTCCCTGCGGGTAAAGATCT
		reverse	pTCCCTTTATTCTTTGCGCGCTCGAAGGAGGCAACGA
fmt	MTF	forward	pAATGTCAGAATCACTACGTATTATTTTT
		reverse	pTCCCGACCAGACGGTTGCCCGG
alaS	AlaRS	forward	pAATGAGCAAGAGCACCGCTGAGATCCGTCAGGCGTT
		reverse	pTCCCTTGCAATTTCGCGCTGACCCAGCCTTTCACAC
argS	ArgRS	forward	pAATGAATATTCAGGCTCTTCTCTCAGAAAAAGTC
		reverse	pTCCCCATACGCTCTACAGTCTCAATACCCAGCGTAT
asnS	AsnRS	forward	pAATGAGCGTTGTGCCTGTAGCCGACGTACTC
		reverse	pTCCCGAAGCTGGCGTTACGCGGAGTACGTGGGAACG
aspS	AspRS	forward	pAATGCGTACAGAATATTGTGGACAGCTCCGT
		reverse	pTCCCGTTATTCTCAGCCTTCTTCACAACCTGAAT
cysS	CysRS	forward	pAATGCTAAAAATCTTCAATACTCTGACACGCCA
		reverse	pTCCCCTTACGACGCCAGGTGGTCCCTTGCGGGCCATCTT
glnS	GlnRS	forward	pAATGAGTGAGGCAGAAGCCCGCCCGACTAACTT
		reverse	pTCCCCTCGCCTACTTTCGCCCAGGTATCACGCAGCCCA
gltX	GluRS	forward	pAATGAAAATCAAAACTCGCTTCGCGCCAAGCCCAAC
		reverse	pTCCCCTGCTGATTTTCGCGTTCAGCAATAAAATCCAGCGCTTTG

Gene	Protein	Primer	
glyQ & glyS	GlyRS	forward	pAATGCAAAAGTTTGATACCAGGACC
		reverse	pTCCCTTGCAACAGCGAAATATCCGC
hisS	HisRS	forward	pAATGGCAAAAAACATTCAAGCCATTC
		reverse	pTCCCACCCAGTAACGTGCGCAAAT
ileS	IleRS	forward	pAATGAGTGACTATAAATCAACCCTGAATTTGCC
		reverse	pTCCCGGCAAACTTACGTTTTTCACCGTCACCGGCGA
leuS	LeuRS	forward	pAATGCAAGAGCAATACCGCCCGGAAGAGATA
		reverse	pTCCCGCCAACGACCAGATTGAGGAGTTTACCTGGTACG
lysS	LysRS	forward	pAATGTCTGAACAACACGCACAGGGCGCTGACGCGGT
		reverse	pTCCCTTTTACCGGACGCATCGCCGGGAACAGAATAA
metG	MetRS	forward	pAATGACTCAAGTCGCGAAGAAAATTCTGGTGACGT
		reverse	pTCCCTTTCACCTGATGACCCGGTTTAGCACC
pheS & pheT	PheRS	forward	pAATGTCACATCTCGCAGAACTGGTTGCCAGTGCGAA
		reverse	pTCCCATCCCTCAATGATGCCTGGAATCGCTCTTTTA
proS	ProRS	forward	pAATGCGTACTAGCCAATACCTGCTCTCCACT
		reverse	pTCCCGCCTTTAATCTGTTTCACCAGATATTCGACGATG
serS	SerRS	forward	pAATGCTCGATCCCAATCTGCTGCGTAATGAGCCAGA
		reverse	pTCCCGCCAATATATTCCAGTCCGTTCATATACGGAC
thrS	ThrRS	forward	pAATGCCTGTTATAACTCTTCCTGATGGCAGC
		reverse	pTCCCTTCCTCCAATTGTTTAAGACTGCGGCTGCGAA
trpS	TrpRS	forward	pAATGACTAAGCCCATCGTTTTTAGTGGCGCAC
		reverse	pTCCCCGGCTTCGCCACAAAACCAATCGCTTCGTACA
tyrS	TyrRS	forward	pAATGGCAAGCAGTAACTTGATTAAACAATTG
		reverse	pTCCCTTTCCAGCAAATCAGACAGTAATTCTTTTT
valS	ValRS	forward	pAATGGAAAAGACATATAACCCACAAGATATCG
		reverse	pTCCCCAGCGCGGCGATAACAGCCTGCTGTTCAATCA

Synthesis of Flexizymes and tRNA. Flexizymes aFx, dFx and eFx (6) and 5′-phosphorylated tRNAs were synthesized on an ÄKTA oligopilot 10 instrument (GE Healthcare) by standard phosphoramidite-chemistry using 2′-TBDMS-protected building blocks. Sequences of the tRNAs (anticodons underlined):

tRNA$^{Gly}_{u}$: pGCGGGCGUAGUUCAAUGGUAGAACGAGAGCUUCCCAAGCUCUAUACGAGGGUUCGAUUCCCUUCGCCCGCUCCA

tRNA$^{Gly}_{tp}$: pGCGGGCGUAGUUCAAUGGUAGAACGAGAGCUUCCCAAGCUCUAUACGAAGGUUCGAUUCCUUCCGCCCGCUCCA

tRNA$^{Tyr}_{u}$: pGGUGGGGUUCCCGAGCGGCCAAAGGGAGCAGACUGUAAAUCUGCCGUCAUCGACUUCGAAGGUUCGAAUCCUUCCCCCACCACCA

tRNA$^{Tyr}_{u\ UCA}$: pGGUGGGGUUCCCGAGCGGCCAAAGGGAGCAGACUUCAAAUCUGCCGUCAUCGACUUCGAAGGUUCGAAUCCUUCCCCCACCACCA

tRNA$^{Tyr}_{tp}$: pGGUGGGGUUCCCGAGCGGCCAAAGGGAGCAGACUGUAAAUCUGCCGUCAUCGACUUCGCGGGUUCGAAUCCCGUCCCCACCACCA

tRNA$^{Tyr}_{tp\ UCA}$: pGGUGGGGUUCCCGAGCGGCCAAAGGGAGCAGACUUCAAAUCUGCCGUCAUCGACUUCGCGGGUUCGAAUCCCGUCCCCACCACCA

Native *E. coli* tRNA^{Tyr} was purchased from Sigma.

Assembly of native tRNA^{Gly}

Due to the special and mutually incompatible deprotection requirements of 4-thiouridine and dihydrouridine, native tRNA^{Gly} was synthesized in three fragments, which were then ligated in two separate steps. Separate ligation steps were required to prevent an otherwise observed direct ligation of fragment 1 to fragment 3.

Fragment 1: pGCGGGCG**4**A (4: 4-thiouridine)

Fragment 2: pGUUCAAUGG**D**A (D: dihydrouridine)

Fragment 3: pGAACGAGAGCUUCCCAAGCUCUAUACGAGGG**TΨ**CGAUUCCCUUCGCCCGCUCCA
(T: ribothymidine; Ψ: pseudouridine)

Synthesis of native tRNA^{Gly} fragment 1: Synthesis was commenced on 0.32g 1000A rA(Pac) CPG (41µmol/g). For coupling rG(Pac), rA(Pac) were used instead of rG(ibu) and rA(bz) amidites. The quantitative coupling of the 4-thiouridine building block was achieved by double coupling of the amidite. Oxidization of phosphit triesters was achieved using 0.02M iodine in pyridine/H_2O (9/1, v/v). Tac_2O served as capping reagent (Proligo fast deprotection capping reagent). After completed oligonucleotide assembly, the CPG was transferred into a glass bottle and dried under reduced pressure. For the removal of cyanoethyl protective groups 10 ml 1M DBU in ACN was added to the dry support at RT. After 2h the supernatant was discarded and the residue CPG was washed 5x with 20 ml ACN. To facilitate cleavage of the oligonucleotide from the CPG 15 ml of 50mM NaSH solution in $tBuNH_2/MeOH/H_2O$ (1/1/2, v/v/v) were added and agitated for 3h at 55°C. After cooling, the supernatant was purified by size exclusion chromatography using NAP25 columns (GE-Healthcare) according to the manufacturer's instructions. The product containing fractions were pooled and freeze dried. Purification was done with IEX-chromatography Source15Q (GE-Healthcare) using 25 mM Tris buffer pH 7.5, 10 %ACN, 2M NaCl at 55°C followed by size exclusion chromatography (NAP10, GE-Healthcare). Yield: 60 OD, 2.40 mg, 798nmol.

Synthesis of native tRNA^{Gly} fragment 2: Synthesis was commenced on 0.32g 1000A rA(Pac) CPG (41µmol/g). For coupling rG(Pac), rA(Pac) were used instead of rG(ibu) and rA(bz) amidites. The quantitative introduction of the dihydrouridine building block was achieved by double coupling of the amidite. Oxidization of phosphit triesters was achieved using 0.05M iodine in pyridine/H_2O (9/1, v/v). Tac_2O (Proligo fast deprotection capping reagent) was used as a capping reagent. After completed oligonucleotide assembly, the CPG was washed for 10 min with 10% Et_2NH in ACN followed by a thorough 10 min wash with ACN for removal of the cyanoethyl protective goups. Finally the CPG was transferred into a glass bottle and dried under reduced pressure. To facilitate cleavage of the oligonucleotide from the CPG 20 ml of 50mM NaSH solution in 28% NH_3 (aq.) were added and agitated for 4h at 25°C. The supernatant was collected, concentrated and purified by size exclusion chromatography using NAP25 columns (GE-Healthcare) according to the manufacturer's instructions. The product containing fractions were pooled and freeze dried. Purification was done with IEX-chromatography Source15Q (GE-Healthcare) using 25 mM Tris buffer pH 7.5, 10 %ACN, 2M NaCl at 55°C followed by size exclusion chromatography (NAP10, GE-Healthcare). Yield: 35 OD, 1.40 mg, 392 nmol.

Synthesis of native tRNA^{Gly} fragment 3: Synthesis was carried out using standard conditions as described above.

Ligation step 1: Triplicates of 200 µl of 10x T4 RNA Ligase 2 buffer (NEB), 15.51 nmol each of tRNA fragment 2 (nucleotides 10-20), fragment 3 (nucleotides 21-76), a complementary DNA strand spanning nucleotides 1-40 (sequence: AGCTTGGGAAGCTCTCGTTCTACCATTGAACTACGCCCGC) and H_2O to 1973 µl were mixed and spread into 30 equal aliquots. The oligonucleotides were annealed by denaturing for 3 minutes at 95°C and

cooling to 4°C at a rate of 0.2°C per second in a PCR cycler. To each aliquot, 30 µg T4 RNA ligase 2 per 1 nmol of fragment 3 were added (total reaction volume 2 ml). The reactions were incubated for 2 h at 37°C and precipitated by the addition of 0.1 volume of 3 M NaOAc pH 5.5 and 2.5 volumes of ethanol and subsequent centrifugation for 15 minutes at 21,500 x g. All pellets were dissolved in a total of 200 µl of 8 M urea and applied to an 8 % TBE-Urea maxi gel. Bands were visualized by UV shadowing. The band of the ligation product was cut out, recovered by electroelution and desalted over a NAP10 column (GE healthcare). This procedure was executed twice, yielding ~9.41 nmol of ligation product (~10%).

Ligation step 2: 150 µl of 10x T4 RNA Ligase buffer (NEB), 9.41 nmol of ligated fragment (2+3), 18.82 nmol of fragment 1, H_2O to 1.5 ml were mixed and spread into eight equal aliquots of 185 µl. Annealing was done as decribed above. To each aliquot, 8 µg of T4 RNA ligase 2 per nmol of fragment 2+3 were added. The reactions were incubated for 2h at 37°C. Precipitation and purification of the ligation product was performed as described above. The final overall yield was ~520 pmol (~1.1%).

Judged by TBE-Urea-PAGE, the yield of both ligation reactions was about 90% (step 1) and 50% (step 2), but the recovery from the purification procedure was poor. Due to the low overall yield, native $tRNA^{Gly}$ could not be used for routine experiments.

Synthesis of (D)-aa-Flexizyme substrates

Synthesis of Flexizyme substrates was commenced from commercially available (D)-amino acid species. If necessary, α-amino- as well as side chain protective groups were introduced by known protocols (7-9). Amino acid building blocks were then reacted to form ABT, DBE or CME esters. As amino acids can undergo racemization upon activation, the reaction conditions have to be selected carefully (10). In most cases, PyBOP (11,12) was used as coupling reagent. For the condensation of Boc-(D)-Asn-OH and H-ABT(Boc), EDC/HOBt was used. H-(D)-Met-DBE was not made by *in-situ* activation, but by reaction with dinitrobenzyl chloride. Cleavage of acid labile protecting groups was done with HCl in Dioxane/CH_2Cl_2. H-(D)-Trp-CME was purchased from Iris Biotech GmbH.

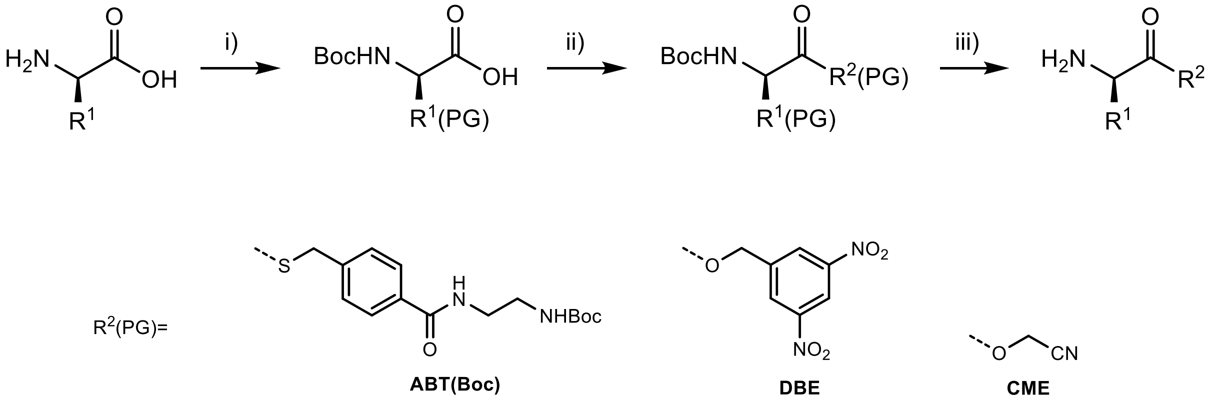

Synthesis of Flexizyme Substrates. i) Boc_2O, KOH or Boc-ON, ii) (Thio)esterification A-C, iii) HCl, CH_2Cl_2, Dioxan, H_2O

The enantiopurity of the final products was determined by C.A.T. GmbH. The protective group pattern, the promoter group introduced to each amino acid and esterification method used as well as the enantiopurity of the final product is given in the table below.

Chemical synthesis procedures

Chemicals were obtained from commercial suppliers and used without further purification unless otherwise noted. PyBOP was purchased from Novabiochem. EDC was from Acros. HOBt, DMF (extra dry), 4M HCl in Dioxane were from Aldrich. Free and eventually N-Boc and/or side chain protected D-amino acid building

blocks were purchased from Bachem and Iris Biotech. 1H- and 13C NMR-spectra were recorded on Bruker DPX 300 or Avance II 500 spectrometer. Chemical shifts (δ, ppm) for ^{1}H and ^{13}C are referenced to internal solvent resonances and reported relative to TMS. TLC was carried out on Merck DC Kieselgel 60 F254 aluminium sheets. Compounds were visualized under short-wavelength UV, with ninhydrin solution (300 mg ninhydrin, 3 ml acetic acid, 97 mL n-butanol) or Seebach-Reagent (2.5 g Molybdophosphoric acid, 1 g Ammonium cerium(IV) sulfate, 6 ml conc. sulfuric acid, 94 ml H_2O). Flash column chromatography was carried out on Kieselgel 60 0.040–0.063 mm (Merck).

Synthesis strategy and enantiopurity of D-aa Flexizyme substrates

Flexyzime Substrate	Side Chain PG	R^2	Esterification Method	Enantiopurity
H-(D)-Ala-ABT	n. a.	ABT	A	>99.9% (<0.1% L-Ala)
H-(D)-Arg-ABT	2xBoc	ABT	A	99.9% (0.1% L-Arg)
H-(D)-Asn-ABT	n. a.	ABT	B	99.76% (0.24% L-Asn)
H-(D)-Asp-ABT	O*t*Bu	ABT	A	99.19% (0.81% L-Asp)
H-(D)-Cys-ABT	Trt	ABT	A	98.98% (1.12% L-Cys); not used
H-(D)-Glu-ABT	O*t*Bu	ABT	A	>99.9% (<0.1% L-Gln)
H-(D)-Gln-ABT	n. a.	ABT	A	99.77% (0.23% L-Glu)
H-(D)-His-ABT	Boc	ABT	A	99.65% (0.35% L-His)
H-(D)-Ile-ABT	n. a.	ABT	A	>99.8% (1.86% D-allo-Ile, <0.1% L-Ile, <0.1% L-allo-Ile)
H-(D)-Leu-ABT	n. a.	ABT	A	99.9% (0.1% L-Leu)
H-(D)-Lys-ABT	Boc	ABT	A	>99.9% (<0.1% L-Lys)
H-(D)-Met-DBE	n. a.	DBE	C	99.83% (0.17% L-Met)
H-(D)-Phe-ABT	n. a.	ABT	A	99.54% (0.46% L-Phe)
H-(D)-Pro-ABT	n. a.	ABT	A	99.82% (0.18% L-Pro)
H-(D)-Ser-ABT	*t*Bu	ABT	A	99.56% (0.44% L-Ser)
H-(D)-Thr-ABT	*t*Bu	ABT	A	>99.8% (0.12% D-allo-Thr, <0.1% L-Thr, <0.1% L-allo-Thr)
H-(D)-Trp-CME	n. a.	CME	-	99.04% (0.96% L-Trp)
H-(D)-Tyr-ABT	Boc	ABT	A	99.84% (0.16% L-Tyr)
H-(D)-Val-ABT	n. a.	ABT	A	>99.9% (<0.1% L-Val)
A) PyBOP, H-ABT(Boc); B) EDC/HOBt, H-ABT(Boc); C) NEt_3, DBE-Cl;				

General Procedure 1 (Thioesterification): At room temperature the Boc- and eventually side chain protected amino acid was dissolved in DMF (0.1 M), 0.95 eq. PyBOP and 2 eq. DÌPEA were added. After 2 min, 1 eq. H-ABT(Boc) was added and the resulting mixture was stirred for 30 min at room temperature. The reaction was quenched by addition of aq. sat. $NaHCO_3$ solution and extracted with EtOAc (3x). The organic phase was washed with water (2x), brine (1x), dried over Na_2SO_4 and concentrated to dryness. Purification by flash column chromatography yielded the desired product.

General Procedure 2 (Boc-cleavage): At room temperature the Boc- and eventually side chain protected amino acid ABT thioester was dissolved in DCM (2 ml/mmol) and 4 M HCl in dioxane (abs.) was added. After stirring for 2h, the reaction was concentrated to dryness and co-evaporated with toluene two times.

Synthesis of Boc-(D)-Ala-ABT(Boc):
Following general procedure 1, 378 mg (2.00 mmol) Boc-(D)-Ala-OH was reacted with 988 mg (1.90 mmol) PyBOP, 680 µl (4.00 mmol) DIPEA and 621 mg (2.00 mmol) H-ABT(Boc) in 20 ml DMF. Yield: 800 mg (0.831 mmol, 43.7 %), off-white solid. Flash column chromatography: Hexane/EtOAc (6/4, v/v), R_f: 0.42 (Hexan/EtOAc, 1/3, v/v). ^{1}H-NMR (DMSO-d6) δ (ppm): 1.21 (3H, m, βCH_3), 1.37 (18H, m, 2*t*Bu), 3.08 (2H, m, N-CH_2-), 3.26 (2H, m, N-CH_2-), 4.10 (3H, m, -S-CH_2, αCH), 6.90 (1H, t, BocNH), 7.33 (2H, d, 2ArCH), 7.67 (1H, d, BocNH), 7.75 (2H, d, 2ArCH), 8.41 (1H, t, Amid-NH). ^{13}C-NMR (DMSO-d6) δ (ppm): 17.6 (βCH_3), 28.6, 28.7 (2*t*Bu), 31.9 (S-CH_2), 56.6 (αCH), 79.1, 78.1 (2C_q), 127.8, 128.9 (2x2ArCH), 133.7, 141.6 (2ArC_q), 155.6, 156.2 (2BocC_q), 166.4 (AmidC_q), 203.1 (Thioester-C_q).

Synthesis of H-(D)-Ala-ABT × 2 HCl:
Following general procedure 2, 833 mg (1.73 mmol) Boc-(D)-Ala-ABT(Boc) dissolved in 3.3 ml DCM was treated with 6.6 mL 4M HCl in Dioxane (abs.) to yield 614 mg (1.73 mmol) H-(D)-Ala-ABT as a colorless solid. ^{1}H-NMR (DMSO-d6) δ (ppm): 1.44 (3H, d, βCH_3), 2.96 (2H, m, N-CH_2-), 3.52 (2H, m, N-CH_2-), 4.28 (3H, m, -S-CH_2, αCH), 7.41 (2H, d, 2ArCH), 7.91 (2H, d, 2ArCH), 8.31 (3H, br, NH_3^+), 8.74 (3H, br, NH_3^+), 8.93 (1H, m, Amid-NH). ^{13}C-NMR (DMSO-d6) δ (ppm): 17.4 (βCH_3), 32.3 (S-CH_2), 37.5, 38.9 (2N-CH_2-), 54.8 (αCH), 128.2, 129.1 (2x2ArCH), 133.4, 141.0 (2ArC_q), 166.6 (AmidC_q), 197.2 (Thioester-C_q).

Synthesis of Boc-(D)-Arg(Boc)$_2$-ABT(Boc):
Following general procedure 1with minor modifications, 1.00 g (2.11 mmol) Boc-(D)-Arg(Boc)$_2$-OH was reacted with 1.10 g (2.11 mmol) PyBOP, 735 µl (4.22 mmol) DIPEA and 586 mg (1.90 mmol) H-ABT(Boc) in 21 mL DMF. Yield: 570 mg (0.734 mmol, 39.3 %), off-white foam. Flash column chromatography: Toluene/Acetone (7/3, v/v), R_f: 0.43 (Hexan/EtOAc, 1/3, v/v). ^{1}H-NMR (DMSO-d6) δ (ppm): 1.37 (27H, m, 3*t*Bu), 1.47 (9H, m, *t*Bu), 1.53 (3H, m, βCHH', γCH_2), 1.69 (1H, m, βCHH'), 3.08 (2H, m, N-CH_2-), 3.27 (4H, m, N-CH_2-, δCH_2), 4.08 (3H, m, -S-CH_2, αCH), 6.90 (1H, t, BocNH), 7.33 (2H, d, 2ArCH), 7.67 (1H, d, BocNH), 7.75 (2H, d, 2ArCH), 8.29 (1H, t, -NH-), 8.41 (1H, t, Amid-NH), 11.51 (1H, s, BocNH). ^{13}C-NMR (DMSO-d6) δ (ppm): 25.7 (γCH_2), 27.8 (βCH_2), 28.1, 28.4, 28.6, 28.7 (4*t*Bu), 32.0 (S-CH_2), 61.0 (αCH), 78.1, 78.5, 79.1, 83.3 (4C_q), 127.8, 128.9 (2x2ArCH), 133.7, 141.5 (2ArC_q), 152.5, 155.7, 155.9, 156.2 (2BocC_q), 163.6 (C_q) 166.4 (AmidC_q), 202.4 (Thioester-C_q).

Synthesis of H-(D)-Arg-ABT × 3 HCl:
Following general procedure 2, 100 mg (0.129 mmol) Boc-(D)-Arg(Boc)$_2$-ABT(Boc) dissolved in 260µL DCM was treated with 515 µl 4M HCl in Dioxane (abs.) to yield 51 mg (0.107 mmol) H-(D)-Arg-ABT as a colorless solid. ^{1}H-NMR (DMSO-d6) δ (ppm): 1.63 (2H, m, γCH_2), 1.69 (2H, m, βCH_2), 2.97 (2H, m, N-CH_2-), 3.25 (2H, m, δCH_2), 3.49 (2H, m, N-CH_2-), 4.29 (3H, m, -S-CH_2, αCH), 7.41 (2H, d, 2ArCH), 7.92 (2H, d, 2ArCH), 7.93 (1H, t, -NH-) 8.31 (3H, br, NH_3^+), 8.81 (3H, br, NH_3^+), 8.95 (1H, m, Amid-NH). ^{13}C-NMR (DMSO-d6) δ (ppm): 24.5 (γCH_2), 28.7 (βCH_2), 32.5 (S-CH_2), 58.2 (αCH), 40.3 (δCH_2), 128.3, 129.1 (2x2ArCH), 133.4, 140.9 (2ArC_q), 157.6 (C_q) 166.7 (AmidC_q), 196.5 (Thioester-C_q).

Synthesis of Boc-(D)-Asn-ABT(Boc):
At room temperature the 339 mg (1.46 mmol) Boc-(D)-Asn-OH and 198 mg (1.29 mmol) HOBt were dissolved in 17 ml DMF. Subsequently 266 mg (1.39 mmol) EDC followed by 500 mg (1.61 mmol) H-ABT(Boc) were added. After 1 hour at room temperature, the reaction mixture was distributed between EtOA and aq. sat. $NaHCO_3$ solution and extracted with EtOAc (3x). The combined organic phase was washed with 0.1 M HCl (1x) and brine (1x), dried over Na_2SO_4, filtered and concentrated to dryness. Purification by flash column

chromatography yielded the desired product. Yield: 205 mg (0.391 mmol, 30.3 %), faint yellow solid. Flash column chromatography: EtOAc, R_f: 0.25 (EtOAc). ^{1}H-NMR (DMSO-d6) δ (ppm): 1.37 (18H, m, 2*t*Bu), 2.47 (2H, m, βCH_2) 3.08 (2H, m, N-CH_2-), 3.26 (2H, m, N-CH_2-), 4.09 (2H, m, -S-CH_2), 4.45 (1H, m, αCH), 6.90 (1H, t, BocNH), 6.96 (1H, s, NHH'), 7.34 (3H, m, 2ArCH, NHH'), 7.51 (1H, d, BocNH), 7.80 (2H, d, 2ArCH), 8.41 (1H, t, Amid-NH). ^{13}C-NMR (DMSO-d6) δ (ppm): 28.6, 28.7 (2*t*Bu), 32.1 (S-CH_2), 37.1 (βCH_2), 57.8 (αCH), 78.1, 79.2 (2C_q), 127.8, 128.9 (2x2ArCH), 133.7, 141.5 (2ArC_q), 155.4, 156.2 (2BocC_q), 166.4, 171.2 (2AmidC_q), 201.8 (Thioester-C_q).

Synthesis of H-(D)-Asn-ABT × 2 HCl:
Following general procedure 2, 205 mg (0.391 mmol) Boc-(D)-Asn-ABT(Boc) dissolved in 780 µl DCM was treated with 1.56 mL 4M HCl in Dioxane (abs.) to yield 156 mg (0.391 mmol) H-(D)-Asn-ABT as a colorless solid. ^{1}H-NMR (DMSO-d6) δ (ppm): 2.85 (2H, m, βCH_2), 2.96 (2H, m, N-CH_2-), 3.52 (2H, m, N-CH_2-), 4.28 (H2, m, -S-CH_2), 4.45 (1H, m, αCH), 7.26 (1H, s, NHH'), 7.40 (2H, d, 2ArCH), 7.80 (1H, s, NHH'), 7.91 (2H, d, 2ArCH), 8.28 (3H, br, NH_3^+), 8.63 (3H, br, NH_3^+), 8.92 (1H, m, Amid-NH). ^{13}C-NMR (DMSO-d6) δ (ppm): 32.3 (S-CH_2), 35.6 (βCH_2), 37.6, 38.9 (2N-CH_2-) 55.7 (αCH), 128.1, 129.1 (2x2ArCH), 133.4, 141.0 (2ArC_q), 166.6 (AmidC_q), 170.2 (AmidC_q), 195.7 (Thioester-C_q).

Synthesis of Boc-(D)-Asp(O*t*Bu)-ABT(Boc):
Following general procedure 1, 578 mg (2.00 mmol) Boc-(D)-Asp(O*t*Bu)-OH was reacted with 988 mg (1.90 mmol) PyBOP, 680 µl (4.00 mmol) DIPEA and 621 mg (2.00 mmol) H-ABT(Boc) in 20 ml DMF. Yield: 552 mg (0.949 mmol, 49.9 %), brown solid. Flash column chromatography: Hexane/EtOAc (6/4, v/v), R_f: 0.48 (Hexan/EtOAc, 1/3, v/v). ^{1}H-NMR (DMSO-d6) δ (ppm): 1.37 (27H, m, 3*t*Bu), 2.46 (1H, dd, βCHH'), 2.74 (1H, dd, βCHH'), 3.08 (2H, m, N-CH_2-), 3.27 (2H, m, N-CH_2-), 4.10 (2H, m, -S-CH_2), 4.47 (1H, m, αCH), 6.90 (1H, t, BocNH), 7.35 (2H, m, 2ArCH), 7.67 (1H, d, BocNH), 7.75 (2H, d, 2ArCH), 8.41 (1H, t, Amid-NH). ^{13}C-NMR (DMSO-d6) δ (ppm): 28.1, 28.6, 28.7 (3*t*Bu), 32.3 (S-CH_2), 37.6 (βCH_2), 57.7 (αCH), 78.1, 79.3, 80.9 (3C_q), 127.8, 129.0 (2x2ArCH), 133.8, 141.4 (2ArC_q), 155.4, 156.2 (2BocC_q), 166.4 (AmidC_q), 169.3 (ester-C_q), 201.8 (Thioester-C_q).

Synthesis of H-(D)-Asp-ABT × 2 HCl:
Following general procedure 2, 552 mg (0.949 mmol) Boc-(D)-Asp(O*t*Bu)-ABT(Boc) dissolved in 1.90 ml DCM was treated with 3.80 ml 4M HCl in Dioxane (abs.) to yield 388 mg (0.974 mmol) H-(D)-Asp-ABT as off-white foam. ^{1}H-NMR (DMSO-d6) δ (ppm): 2.98 (4H, m, βCH_2, N-CH_2-), 3.52 (2H, m, N-CH_2-), 4.29 (H2, m, -S-CH_2), 4.45 (1H, m, αCH), 7.40 (2H, d, 2ArCH), 7.90 (H, d, 2ArCH), 8.26 (3H, br, NH_3^+), 8.75 (3H, br, NH_3^+), 8.91 (1H, t, Amid-NH). ^{13}C-NMR (DMSO-d6) δ (ppm): 32.4 (S-CH_2), 35.6 (βCH_2), 37.6, 38.9 (2N-CH_2-) 55.4 (αCH), 128.2, 129.1 (2x2ArCH), 133.4, 140.9 (2ArC_q), 166.6 (AmidC_q), 170.8 (-COOH), 195.3 (Thioester-C_q).

Synthesis of Boc-(D)-Cys(Trt)-ABT(Boc):
Following general procedure 1 with minor modifications, 1.39 g (3.00 mmol) Boc-(D)-Cys(Trt)-OH was reacted with 1.48 g (2.85 mmol) PyBOP, 1.02 ml (6.00 mmol) DIPEA and 0.621 mg (2.00 mmol) H-ABT(Boc) in 20 ml DMF. Yield: 1.22 g (1.61 mmol, 80.6 %), colorless solid. Flash column chromatography: Hexane/EtOAc (3/2, v/v), R_f: 0.66 (Hexane/EtOAc, 1/3, v/v). ^{1}H-NMR (DMSO-d6) δ (ppm): 1.40 (18H, m, 2*t*Bu),2.36 (1H, m, βCHH'), 2.60 (1H, m, βCHH'), 3.10 (2H, m, N-CH_2-), 3.28 (2H, m, N-CH_2-), 3.81 (1H, m, αCH), 4.01 (2H, s, -S-CH_2), 6.90 (1H, t, BocNH), 7.30 (17H, m, 17ArCH), 7.73 (3H, m, 2ArCH, BocNH), 8.41 (1H, t, AmidNH). ^{13}C-NMR (DMSO-d6) δ (ppm): 28.6, 28.7 (2*t*Bu), 30.6, 31.5 (CH_2), 32.2 (S-CH_2), 33.1 (βCH_2), 60.5 (αCH), 67.0, 78.1, 79.3 (3C_q), 127.7 (3ArCH), 127.8 (2ArCH), 128.6 (6ArCH), 128.9 (2ArCH), 129.5 (6ArCH), 133.7, 141.2 (2ArC_q), 144.6 (3ArC_q), 155.5, 156.2 (2BocC_q), 166.4 (AmidC_q), 200.3 (Thioester-C_q).

Synthesis of H-(D)-Cys-ABT × 2 TFA:
At room temperature 200 mg (0.264 mmol) Boc-(D)-Cys(trt)-ABT(Boc) were placed in a round bottom flask and a solution of 90µL *i*Pr_3SiH in 2.6 mL TFA was added. Instantly a white solid precipitated. After 2 h, 10 mL Et_2O was added and the resulting precipitate was collected by centrifugation. The sticky residue was dried under reduced pressure to yield 149 mg (0.275 mmol) H-(D)-Cys-ABT as colorless solid. ^{1}H-NMR (DMSO-d6) δ (ppm): ^{1}H-NMR (DMSO-d6) δ (ppm): 2.99 (4H, m, N-CH_2-, βCH_2), 3.50 (2H, m, N-CH_2-), 4.33 (2H, m, -S-CH_2), 4.58 (αCH), 7.44 (2H, d, 2ArCH), 7.83 (2H, d, 2ArCH), 8.03 (3H, br, NH_3^+), 8.68 (4H, br/m, NH_3^+, AmidNH). ^{13}C-NMR (DMSO-d6) δ (ppm): 27.8 (βCH_2), 32.5 (S-CH_2), 37.6, 39.0 (2N-CH_2-), 60.1 (αCH), 128.5 (2ArCH), 129.2 (2ArCH), 133.6, 140.9 (2ArC_q), 166.9 (AmidC_q), 194.9 (Thioester-C_q).

Synthesis of Boc-(D)-Glu(O*t*Bu)-ABT(Boc):
Following general procedure 1, 607 mg (2.00 mmol) Boc-(D)-Glu(O*t*Bu)-OH was reacted with 988 mg (1.90 mmol) PyBOP, 680 µl (4.00 mmol) DIPEA and 621 mg (2.00 mmol) H-ABT(Boc) in 20 mL DMF. Yield: 630 mg (1.06 mmol, 55.7 %), brown solid. Flash column chromatography: Hexane/EtOAc (6/4, v/v), R_f: 0.40 (Hexan/EtOAc, 1/3, v/v). ^{1}H-NMR (DMSO-d6) δ (ppm): 1.37 (27H, m, 3*t*Bu), 1.67 (1H, dd, βCHH'), 1.92 (1H, dd, βCHH'), 2.25 (2H, m, γCH_2), 3.08 (2H, m, N-CH_2-), 3.26 (2H, m, N-CH_2-), 4.10 (3H, m, -S-CH_2, αCH), 6.90 (1H, t, BocNH), 7.35 (2H, m, 2ArCH), 7.63 (1H, d, BocNH), 7.74 (2H, d, 2ArCH), 8.41 (1H, t, Amid-NH). ^{13}C-NMR (DMSO-d6) δ (ppm): 26.6 (βCH_2), 28.2, 28.6, 28.7 (3*t*Bu), 31.3 (γCH_2), 32.0 (S-CH_2), 60.0 (αCH), 78.1, 79.2, 80.3 (3C_q), 127.8, 128.9 (2x2ArCH), 133.7, 141.5 (2ArC_q), 155.9, 156.2 (2BocC_q), 166.4 (AmidC_q), 171.8 (ester-C_q), 202.2 (Thioester-C_q).

Synthesis of H-(D)-Glu-ABT × 2 HCl:
Following general procedure 2, 630 mg (1.06 mmol) Boc-(D)-Glu(O*t*Bu)-ABT(Boc) dissolved in 1.00 mL DCM was treated with 2.00 ml 4M HCl in Dioxane (abs.) to yield 530 mg (1.28 mmol) H-(D)-Glu-ABT as a brown solid. ^{1}H-NMR (DMSO-d6) δ (ppm): 2.03 (2H, m, γCH_2), 2.34 (1H, m, βCHH'), 2.48 (1H, m, βCHH'), 2.97 (2H, m, N-CH_2-), 3.52 (2H, m, N-CH_2-), 4.29 (3H, m, -S-CH_2, αCH), 7.42 (2H, d, 2ArCH), 7.91 (H, d, 2ArCH), 8.26 (3H, br, NH_3^+), 8.78 (3H, br, NH_3^+), 8.89 (1H, t, Amid-NH). ^{13}C-NMR (DMSO-d6) δ (ppm): 27.0, 29.3 (βCH_2, γCH_2) 32.5 (S-CH_2), 37.6, 38.9 (2N-CH_2-) 57.9 (αCH), 128.2, 129.1 (2x2ArCH), 133.5, 140.9 (2ArC_q), 166.6 (AmidC_q), 173.5 (-COOH), 196.3 (Thioester-C_q).

Synthesis of Boc-(D)-Gln-ABT(Boc):
Following general procedure 1, 500 mg (2.03 mmol) Boc-(D)-Gln-OH was reacted with 1.00 g (1.92 mmol) PyBOP, 692 µl (4.06 mmol) DIPEA and 630 mg (2.03 mmol) H-ABT(Boc) in 20 ml DMF. Yield: 490 mg (0.910 mmol, 47.4 %), faint yellow solid. Flash column chromatography: EtOAc, EtOAc/MeOH (9/1, v/v), R_f: 0.55 (EtOAc/MeOH, 9/1, v/v). ^{1}H-NMR (DMSO-d6) δ (ppm): 1.37 (18H, m, 2*t*Bu), 1.67 (1H, dd, βCHH'), 1.92 (1H, dd, βCHH'), 2.13 (2H, m, γCH_2), 3.08 (2H, m, N-CH_2-), 3.26 (2H, m, N-CH_2-), 4.07 (3H, m, -S-CH_2, αCH), 6.78 (1H, s, NHH'), 6.90 (1H, t, BocNH), 7.28 (1H, s, NHH'), 7.34 (2H, m, 2ArCH), 7.65 (1H, d, BocNH), 7.75 (2H, d, 2ArCH), 8.41 (1H, t, Amid-NH). ^{13}C-NMR (DMSO-d6) δ (ppm): 27.0 (βCH_2), 28.6, 28.7 (2*t*Bu), 31.4 (γCH_2), 31.9 (S-CH_2), 60.7 (αCH), 78.1, 79.1 (C_q), 127.8, 128.9 (2x2ArCH), 133.7, 141.5 (2ArC_q), 155.9, 156.2 (2BocC_q), 166.5 (AmidC_q), 173.7 (AmidC_q), 202.4 (Thioester-C_q).

Synthesis of H-(D)-Gln-ABT × 2 HCl:
Following general procedure 2 with minor modifications, 490 mg (0.909 mmol) Boc-(D)-Gln-ABT(Boc) dissolved in 11.0 mL DCM was treated with 7.26 ml 4M HCl in Dioxane (abs.) to yield 387 mg (0.941 mmol) H-(D)-Glu-ABT as a colorless solid. ^{1}H-NMR (DMSO-d6) δ (ppm): 2.01 (2H, m, γCH_2), 2.29 (2H, m, βCH_2), 2.97 (2H, m, N-CH_2-), 3.53 (2H, m, N-CH_2-), 4.18 (1H, m, αCH), 4.29 (2H, m, -S-CH_2), 6.96 (1H, s, NHH'), 7.42 (2H, d, 2ArCH), 7.52 (1H, s, NHH'), 7.92 (2H, d, 2ArCH), 8.29 (3H, br, NH_3^+), 8.80 (3H, br, NH_3^+), 8.92 (1H, t, Amid-NH). ^{13}C-NMR

(DMSO-d6) δ (ppm): 27.5, 30.3 (βCH_2, γCH_2) 32.5 ($S-CH_2$), 37.6, 38.9 ($2N-CH_2$-) 58.2 (αCH), 128.2, 129.1 (2x2ArCH), 133.5, 140.8 ($2ArC_q$), 166.7 ($AmidC_q$), 173.3 (-COOH), 196.4 (Thioester-C_q).

Synthesis of Boc-(D)-His(Boc)-ABT(Boc):

Following general procedure 1, 629 mg (1.77 mmol) Boc-(D)-His(Boc)-OH was reacted with 796 mg (1.52 mmol) PyBOP, 548 µl (3.22 mmol) DIPEA and 500 mg (1.61 mmol) H-ABT(Boc) in 16 mL DMF. Two successive flash column chromatography runs were needed for purification. Yield: 230 mg (0.355 mmol, 23.4 %), colorless foam. 1st flash column chromatography: CH_2Cl_2/MeOH (100/1 to 100/5, v/v), 2nd flash column chromatography: EtOAc/Hexane (2/1, v/v), R_f: 0.50 (CH_2Cl_2/MeOH, 100/5, v/v), 0.71 (EtOAc/Hexane, 10/1, v /v). ^1H-NMR (DMSO-d6) δ (ppm): 1.35 (9H, s, *t*Bu), 1.36 (9H, s, *t*Bu), 1.55 (9H, s, *t*Bu), 2.76 (1H, dd, βCHH'), 1.91 (1H, dd, βCHH'), 3.08 (2H, m, N-CH_2-), 3.26 (2H, m, N-CH_2-), 4.11 (1H, s, -S-CH_2), 4.36 (1H, m, αCH), 6.90 (1H, t, BocNH), 7.23 (1H, s, ArCH), 7.33 (2H, m, 2ArCH), 7.61 (1H, d, BocNH), 7.74 (2H, d, 2ArCH), 8.12 (1H, d, ArCH), 8.41 (1H, t, Amid-NH). ^{13}C-NMR ($CDCl_3$) δ (ppm): 27.9 (*t*Bu) 28.3 (2*t*Bu), 29.7 (βCH_2), 32.9 (S-CH_2), 40.0, 41.8 (2xN-CH_2-), 59.9 (αCH), (βCH_2), 80.1, 80.3, 85.9 ($3C_q$), 114.8 (ArCH), 126.5 (ArC_q), 127.3, 128.9 (2x2ArCH), 132.8, 141.8 (ArC_q), 144.8 (ArCH), 155.4, 155.6, 157.8 ($3BocC_q$), 167.5 ($AmidC_q$), 201.0 (Thioester-C_q).

Synthesis of H-(D)-His-ABT × 3 HCl:

Following general procedure 2 with minor modifications, 230 mg (0.355 mmol) Boc-(D)-His(Boc)-ABT(Boc) dissolved in 710 µl DCM was treated with 1.42 ml 4M HCl in Dioxane (abs.) to yield 159 mg (0.349 mmol) H-(D)-His-ABT as a colorless solid. ^1H-NMR (DMSO-d6) δ (ppm): 2.98 (2H, m, N-CH_2-), 3.30 (2m, d, βCH_2), 3.53 (2H, m, N-CH_2-), 4.27 (2H, m, -S-CH_2), 2.76 (1H, t, αCH), 7.34 (2H, d, 2ArCH), 7.46 (1H, s, ArCH), 7.91 (2H, d, 2ArCH), 8.28 (3H, br, NH_3^+), 8.94 (1H, t, Amid-NH), 9.10 (1H, s, ArCH) ^{13}C-NMR (DMSO-d6) δ (ppm): 26.8 (βCH_2) 32.6 (S-CH_2), 37.6, 38.9 (2N-CH_2-) 57.4 (αCH), 118.8 (ArCH), 126.5 (ArC_q), 128.2, 129.0 (2x2ArCH), 132.2 (ArCH), 133.5, 140.7 ($2ArC_q$), 166.7 ($AmidC_q$), 195.3 (Thioester-C_q).

Synthesis of Boc-(D)-Ile-ABT(Boc):

Following general procedure 1, 910 mg (4.32 mmol) Boc-(D)-Ile-OH was reacted with 2.14 g (4.11 mmol) PyBOP, 1.47 ml (8.65 mmol) DIPEA and 1.34 g (4.32 mmol) H-ABT(Boc) in 43 ml DMF. Yield: 1.70 g (3.25 mmol, 79.0 %), colorless solid. Flash column chromatography: Hexane/EtOAc (3/2 to 1/1, v/v), R_f: 0.66 (Hexane/EtOAc, 3/1, v/v). ^1H-NMR (DMSO-d6) δ (ppm): 0.77 (6H, m, $2CH_3$), 1.20 (2H, m, γCH_2), 1.37 (18H, m, 2*t*Bu), 1.80 (1H, m, βCH), 3.08 (2H, m, N-CH_2-), 3.26 (2H, m, N-CH_2-), 3.95 (1H, m, αCH), 4.10 (2H, s, -S-CH_2) 6.90 (1H, t, BocNH), 7.33 (2H, d, 2ArCH), 7.60 (1H, d, BocNH), 7.75 (2H, d, 2ArCH), 8.41 (1H, t, Amid-NH). ^{13}C-NMR (DMSO-d6) δ (ppm): 11.6, 15.8 ($2CH_3$), 25.1 (γCH_2), 28.7 (2*t*Bu), 31.9 (S-CH_2), 36.5 (βCH), 65.6 (αCH), 78.1, 79.1 ($2C_q$), 127.8, 128.9 (2x2ArCH), 133.6, 141.8 ($2ArC_q$), 156.2 ($2BocC_q$), 166.4 ($AmidC_q$), 201.7 (Thioester-C_q).

Synthesis of H-(D)-Ile-ABT × 2 HCl:

Following general procedure 2, 1.70 g (3.25 mmol) Boc-(D)-Ile-ABT(Boc) dissolved in 6.5 ml DCM was treated with 12.9 ml 4M HCl in Dioxane (abs.) to yield 1.36 g (3.43 mmol) H-(D)-Ile-ABT as off-white foam. ^1H-NMR (DMSO-d6) δ (ppm): 0.86 (6H, m, $2CH_3$), 1.22 (1H, m, γCHH'), 1.39 (1H, m, γCHH'), 1.95 (1H, m, βCH), 2.97 (2H, m, N-CH_2-), 3.52 (2H, m, N-CH_2-), 4.16 (1H, m, αCH), 4.30 (3H, s, -S-CH_2), 7.41 (2H, d, 2ArCH), 7.91 (2H, d, 2ArCH), 8.29 (3H, br, NH_3^+), 8.72 (3H, br, NH_3^+), 8.91 (1H, m, Amid-NH). ^{13}C-NMR (DMSO-d6) δ (ppm): 12.0, 14.8 ($2CH_3$), 25.1 (γCH_2), 32.5 (S-CH_2), 37.0 (βCH_3), 37.6, 38.9 (2N-CH_2-), 62.7 (αCH), 128.2, 129.1 (2x2ArCH), 133.4, 141.0 ($2ArC_q$), 166.6 ($AmidC_q$), 195.7 (Thioester-C_q).

Synthesis of Boc-(D)-Leu-ABT(Boc):

Following general procedure 1, 870 mg (3.76 mmol) Boc-(D)-Leu-OH was reacted with 1.85 g (3.57 mmol) PyBOP, 1.28 ml (7.52 mmol) DIPEA and 1.17 g (3.76 mmol) H-ABT(Boc) in 38 ml DMF. Yield: 1.43 g (2.73 mmol, 76.5 %), colorless solid. Flash column chromatography: Hexane/EtOAc (1/1, v/v), R_f: 0.66 (Hexane/EtOAc, 3/1, v/v). ^{1}H-NMR ($CDCl_3$) δ (ppm): 0.94 (6H, m, 2CH_3), 1.44 (19H, m, 2*t*Bu, γCH_2), 1.69 (2H, m, βCH_2), 3.39 (2H, m, N-CH_2-), 3.53 (2H, m, N-CH_2-), 4.11 (2H, s, -S-CH_2), 4.38 (1H, m, αCH), 4.95 (1H, d, BocNH), 5.15 (1H, br, AmidNH), 7.32 (2H, d, 2ArCH), 7.74 (2H, d, 2ArCH). ^{13}C-NMR ($CDCl_3$) δ (ppm): 23.1 (2CH_3), 24.8 (γCH), 28.3 (2*t*Bu), 32.8 (S-CH_2), 40.0 (N-CH_2-), 41.5 (βCH), 42.0 (N-CH_2-), 59.2 (αCH), 79.9, 80.3 (2C_q), 127.4, 128.9 (2x2ArCH), 133.1, 141.0 (2ArC_q), 155.2, 157.4 (2BocC_q), 167.4 (AmidC_q), 201.5 (Thioester-C_q).

Synthesis of H-(D)-Leu-ABT × 2 HCl:

Following general procedure 2, 1.43 g (2.73 mmol) Boc-(D)-Leu-ABT(Boc) dissolved in 5.5 ml DCM was treated with 10.9 ml 4M HCl in Dioxane (abs.) to yield 1.00 g (2.52 mmol) H-(D)-Leu-ABT as a colorless solid. ^{1}H-NMR (DMSO-d6) δ (ppm): 0.87 (6H, m, 2δCH_3), 1.71 (3H, m, γCH, βCH_2), 2.97 (2H, m, N-CH_2-), 3.53 (2H, m, N-CH_2-), 4.17 (1H, m, αCH), 4.30 (3H, s, -S-CH_2), 7.41 (2H, d, 2ArCH), 7.92 (2H, d, 2ArCH), 8.33 (3H, br, NH_3^+), 8.78 (3H, br, NH_3^+), 8.94 (1H, m, Amid-NH). ^{13}C-NMR (DMSO-d6) δ (ppm): 22.3, 22.8 (2δCH_3), 24.2 (γCH), 32.5 (S-CH_2), 37.6, 38.9 (2N-CH_2-), 40.8 (βCH_3), 57.3 (αCH), 128.2, 129.1 (2x2ArCH), 133.4, 140.9 (2ArC_q), 166.6 (AmidC_q), 196.8 (Thioester-C_q).

Synthesis of Boc-(D)-Lys(Boc)-ABT(Boc):

Following general procedure 1, 346 mg (1.00 mmol) Boc-(D)-Lys(Boc)-OH was reacted with 494 mg (0.95 mmol) PyBOP, 340 µl (2.00 mmol) DIPEA and 310 mg (1.00 mmol) H-ABT(Boc) in 10 ml DMF. Yield: 400 mg (0.626 mmol, 65.9 %), colorless solid. Flash column chromatography: Hexane/EtOAc (7/3 to 1/1, v/v), R_f: 0.54 (Hexane/EtOAc, 1/3, v/v). ^{1}H-NMR (DMSO-d6) δ (ppm): 1.24-1.40 (31H, m, 3*t*Bu, 2CH_2), 1.53 (2H, m, βCH_2), 2.86 (2H, m, εCH_2), 3.08 (2H, m, N-CH_2-), 3.26 (2H, m, N-CH_2-), 3.99 (1H, m, αCH), 4.08 (2H, s, -S-CH_2), 6.77 (1H, t, BocNH), 6.90 (1H, t, BocNH), 7.34 (2H, d, 2ArCH), 7.60 (1H, d, BocNH), 7.74 (2H, d, 2ArCH), 8.40 (1H, t, AmidNH). ^{13}C-NMR (DMSO-d6) δ (ppm): 23.2 (CH_2), 28.6, 28.7, 28.7 (3*t*Bu), 29.4, 30.9 (2CH_2), 31.9 (S-CH_2), 61.2 (αCH), 77.8, 78.1, 79.1 (3C_q), 127.8, 128.9 (2x2ArCH), 133.7, 141.6 (2ArC_q), 156.0, 156.0, 156.2 (3BocC_q), 166.4 (AmidC_q), 202.6 (Thioester-C_q).

Synthesis of H-(D)-Lys-ABT × 3 HCl:

Following general procedure 2, 400 mg (0.630 mmol) Boc-(D)-Lys(Boc)-ABT(Boc) dissolved in 1 ml DCM was treated with 2 ml 4M HCl in Dioxane (abs.) to yield 260 mg (0.581 mmol) H-(D)-Lys-ABT as a brown solid. ^{1}H-NMR (DMSO-d6) δ (ppm): 1.37 (2H, m, βCH_2), 1.57 (2H, m, γCH_2), 1.84 (2H, m, δCH_2), 2.68 (2H, m, εCH_2), 2.97 (2H, m, N-CH_2-), 3.52 (2H, m, N-CH_2-), 4.26 (3H, m, αCH, -S-CH_2), 7.42 (2H, d, 2ArCH), 7.92 (2H, d, 2ArCH), 8.17 (3H, br, NH_3^+), 8.31 (3H, br, NH_3^+), 8.82 (3H, br, NH_3^+), 8.93 (1H, t, AmidNH). ^{13}C-NMR (DMSO-d6) δ (ppm): 21.3 (γCH_2), 26.5 (βCH_2), 30.8 (δCH_2), 32.5 (S-CH_2), 37.6, 38.8 (2N-CH_2-), 58.3 (αCH), 128.2, 129.1 (2x2ArCH), 133.4, 140.9 (2ArC_q), 166.6 (AmidC_q), 196.5 (Thioester-C_q).

Synthesis of Boc-(D)-Met-DBE:

In a 25ml flask, 1.10 g (4.40 mmol) Boc-(D)-Met-OH were dissolved in 5 ml DMF. To this solution 516 µl (7.48 mmol) DIPEA and 810 mg (3.74 mmol) 3,5-dinitrophenyl chloride were added. The reaction was stirred at room temperature. After 16 h, Et_2O (100 mL) and 0.5M HCl (100 ml) were added, the organic phase was separated, washed with 0.1M HCl (2x, 100 ml), dried over Mg_2SO_4 and concentrated under reduced pressure. The crude product was further purified by flash column chromatography (Hexane/EtOAc, 5/1, v/v). Yield: 1.06 g (2.55 mmol, 68.2 %), yellow oil, R_f: 0.26 (Hexane/EtOAc, 5/1, v/v). ^{1}H-NMR (DMSO-d6) δ (ppm): 1.36 (9H, s, *t*Bu), 1.93 (2H, m, βCH_2), 2.03 (3H, s, CH_3), 2.51 (2H, m, γCH_2), 4.20 (1H, m, αCH), 5.41 (2H, s, CH_2), 7.46

(1H, d, BocNH), 8.67 (2H, d, 2ArCH), 8.80 (1H, t, 1ArCH). ^{13}C-NMR (DMSO-d6) δ (ppm): 14.9 (CH_3), 28.5 (*t*Bu), 30.1, 30.5 (2CH_2), 53.1 (αCH), 64.5 (CH_2), 78.9 (C_q), 118.5 (ArC_q), 128.4 (2ArCH), 141.6 (ArC_q), 148.5 (2ArC_q), 156.1 (BocC_q), 172.7 (Ester-C_q).

Synthesis of H-(D)-Met-DBE × HCl:
Following general procedure 2, 200 mg (0.466 mmol) Boc-(D)-Met-DBE dissolved in 960 µl DCM was treated with 1.92 ml 4M HCl in Dioxane (abs.) to yield 180 mg (0.494 mmol) H-(D)-Met-DBE as a colorless foam. ^{1}H-NMR (DMSO-d6) δ (ppm): 2.03 (3H, s, CH_3), 2.16 (2H, m, γCH_2), 2.64 (2H, m, βCH_2), 4.23 (1H, m, αCH), 5.50 (2H, s, CH_2), 8.77 (2H, m, 2ArCH), 8.81 (1H, m, 1ArCH), 8.93 (3H, br, NH_3^+). ^{13}C-NMR (DMSO-d6) δ (ppm): 14.7 (CH_3), 28.9, 29.8 (2CH_2), 51.3 (αCH), 65.5 (CH_2), 118.8 (ArC_q), 129.0 (2ArCH), 140.1 (ArC_q), 148.5 (2ArC_q), 169.3 (Ester-C_q).

Synthesis of Boc-(D)-Phe-ABT(Boc):
Following general procedure 1, 530 mg (2.00 mmol) Boc-(D)-Phe-OH was reacted with 796 mg (1.53 mmol) PyBOP, 548 µl (3.22 mmol) DIPEA and 500 mg (1.61 mmol) H-ABT(Boc) in 16 ml DMF. Yield: 700 mg (1.25 mmol, 82.0 %), colorless solid. Flash column chromatography: Hexane/EtOAc (2/1 to 1/3, v/v), R_f: 0.63 (Hexane/EtOAc, 1/3, v/v). ^{1}H-NMR (DMSO-d6) δ (ppm): 1.30 (18H, m, 2*t*Bu), 2.76 (1H, m, βCHH'), 3.03 (3H, m, N-CH_2-, βCHH'), 3.24 (2H, m, N-CH_2-), 4.08 (2H, s, -S-CH_2), 4.24 (1H, m, αCH), 6.86 (1H, t, BocNH), 7.20 (5H, m, 5ArCH), 7.32 (2H, d, 2ArCH), 7.65 (1H, d, BocNH), 7.73 (2H, d, 2ArCH), 8.39 (1H, t, AmidNH). ^{13}C-NMR (DMSO-d6) δ (ppm): 28.2, 28.6 (2*t*Bu), 32.2 (S-CH_2), 36.9 (βCH_2), 62.7 (αCH), 78.2, 79.2 (2C_q), 127.0 (ArCH), 127.9, 128.7, 129.1, 129.6 (4x2ArCH), 133.8, 137.9, 141.6 (3ArC_q), 155.8, 156.3 (2BocC_q), 166.6 (AmidC_q), 201.8 (Thioester-C_q).

Synthesis of H-(D)-Phe-ABT × 2 HCl:
Following general procedure 2, 350 mg (0.627 mmol) Boc-(D)-Phe-ABT(Boc) dissolved in 1.26 ml DCM was treated with 2.5 ml 4M HCl in Dioxane (abs.) to yield 260 mg (0.581 mmol) H-(D)-Phe-ABT as a brown solid. ^{1}H-NMR (DMSO-d6) δ (ppm): 3.04 (3H, m, N-CH_2-, βCHH'), 3.24 (1, m, βCHH'), 3.54 (2H, m, N-CH_2-), 4.21 (2H, m, -S-CH_2), 4.48 (1H, m, αCH), 7.42 (2H, d, 2ArCH), 7.17 (2H, m, 2ArCH), 7.25 (3H, m, 3ArCH), 7.31 (2H, m, 2ArCH), 7.91 (2H, d, 2ArCH), 8.32 (3H, br, NH_3^+), 8.95 (1H, t, AmidNH). ^{13}C-NMR (DMSO-d6) δ (ppm): 32.5 (S-CH_2), 37.4 (βCH_2), 37.6, 39.0 (2N-CH_2-), 59.7 (αCH), 127.8 (ArCH), 128.1, 129.0, 129.1, 130.0 (4x2ArCH), 133.3, 134.7, 140.8 (3ArC_q), 166.6 (AmidC_q), 196.1 (Thioester-C_q).

Synthesis of Boc-(D)-Pro-ABT(Boc):
Following general procedure 1 with minor modifications, 646 mg (3.00 mmol) Boc-(D)-Pro-OH was reacted with 1.48 g (2.85 mmol) PyBOP, 1.02 ml (6.00 mmol) DIPEA and 0.621 mg (2.00 mmol) H-ABT(Boc) in 20 ml DMF. Yield: 790 mg (1.56 mmol, 77.9 %), colorless solid. Flash column chromatography: Hexane/EtOAc (1/1, v/v), R_f: 0.33 (Hexane/EtOAc, 1/1, v/v). ^{1}H-NMR (2 Rotamers) (DMSO-d6) δ (ppm): 1.20-1.40 (18H, m, 2*t*Bu), 1.81 (3H, m, CH_2, CHH'), 2.22 (1H, m CHH'), 3.08 (2H, m, N-CH_2-), 3.27 (2H, m, N-CH_2-), 3.35 (2H, m, CH_2), 4.14 (2H, m, -S-CH_2), 4.34 (1H, m, αCH), 6.90 (1H, t, BocNH), 7.36 (2H, m, 2ArCH), 7.76 (2H, m, 2ArCH), 8.41 (1H, t, AmidNH). ^{13}C-NMR (2 Rotamers) (DMSO-d6) δ (ppm): 23.5, 24.2 (CH_2), 28.2, 28.5, 28.7 (2*t*Bu), 30.6, 31.5 (CH_2), 31.8, 31.8 (S-CH_2), 46.9, 47.1 (CH_2), 66.1 (αCH), 78.1, 79.8, 79.9 (2C_q), 127.8 (ArCH), 128.8, 128.9 (ArCH), 133.7, 133.7, 141.5, 141.8 (2ArC_q), 153.3, 154.2, 156.2 (2BocC_q), 166.3, 166.4 (AmidC_q), 201.8 (Thioester-C_q).

Synthesis of H-(D)-Pro-ABT × 2 HCl:
Following general procedure 2, 105 mg (0.207 mmol) Boc-(D)-Pro-ABT(Boc) dissolved in 414 µl DCM was treated with 828 µl 4M HCl in Dioxane (abs.) to yield 79 mg (0.207 mmol) H-(D)-Pro-ABT as a colorless solid. ^{1}H-NMR (DMSO-d6) δ (ppm): 1.89 (3H, m, CH_2, CHH'), 2.33 (1H, m CHH'), 2.97 (2H, m, N-CH_2-), 3.19 (2H, m, CH_2), 3.53 (2H, m, N-CH_2-), 4.31 (2H, m, -S-CH_2), 4.59 (1H, m, αCH), 7.42 (2H, m, 2ArCH), 7.92 (2H, m, 2ArCH),

8.33 (3H, br, NH_3^+), 8.93 (1H, t, AmidNH). ^{13}C-NMR (DMSO-d6) δ (ppm): 23.6, 29.5 (2CH_2), 32.6 (S-CH_2), 38.9, 37.5 (2N-CH_2-), 45.6 (CH_2), 65.3 (αCH), 128.2, 129.1 (2x2ArCH), 133.5, 140.8 (2ArC_q), 166.6 (AmidC_q), 195.4 (Thioester-C_q).

Synthesis of Boc-(D)-Ser(tBu)-ABT(Boc):
Following general procedure 1, 523 mg (2.00 mmol) Boc-(D)-Ser(tBu)-OH was reacted with 988 mg (1.90 mmol) PyBOP, 680 µl (4.00 mmol) DIPEA and 621 mg (2.00 mmol) H-ABT(Boc) in 20 ml DMF. Yield: 606 mg (1.09 mmol, 57.6 %), brown solid. Flash column chromatography: Hexane/EtOAc (6/4, v/v), R_f: 0.50 (Hexane/EtOAc, 1/3, v/v). ^{1}H-NMR (DMSO-d6) δ (ppm): 1.08 (9H, s, *t*Bu), 1.38 (18H, m, 2*t*Bu), 3.08 (2H, m, N-CH_2-), 3.26 (2H, m, N-CH_2-), 3.54 (2H, m, βCH_2), 4.11 (2H, s, -S-CH_2), 4.20 (1H, m, αCH), 6.90 (1H, t, BocNH), 7.33 (3H, m, 2ArCH, BocNH), 7.74 (2H, d, 2ArCH), 8.41 (1H, t, Amid-NH). ^{13}C-NMR (DMSO-d6) δ (ppm): 27.6, 28.6, 28.7 (3tBu), 32.0 (-S-CH_2), 61.7 (βCH_2), 61.8 (αCH), 73.4, 78.1, 79.2 (3 C_q), 127.7, 128.9 (2x2ArCH), 133.6, 141.5 (ArC_q), 155.8, 156.2 (2BocC_q), 166.4 (AmidC_q), 200.5 (Thioester-C_q).

Synthesis of H-(D)-Ser-ABT × 2 HCl:
Following general procedure 2, 606 mg (1.09 mmol) Boc-(D)-Ser-ABT(Boc) dissolved in 2.19 ml DCM was treated with 4.37 ml 4M HCl in Dioxane (abs.) to yield 385 mg (1.04 mmol) H-(D)-Ser-ABT as a colorless solid. ^{1}H-NMR (DMSO-d6) δ (ppm): 2.97 (2H, m, N-CH_2-), 3.52 (2H, m, N-CH_2-), 3.88 (2H, m, βCH_2), 4.30 (2H, m, -S-CH_2), 4.36 (1H, m, αCH), 7.42 (2H, d, 2ArCH), 7.91 (2H, d, 2ArCH), 8.27 (3H, br, NH_3^+), 8.64 (3H, br, NH_3^+), 8.91 (1H, t, AmidNH). ^{13}C-NMR (DMSO-d6) δ (ppm): 32.3 (S-CH_2), 37.6, 39.0 (2N-CH_2-), 60.9 (βCH_2), 61.0 (αCH), 128.2, 129.1 (2x2ArCH), 133.4, 141.0 (2ArC_q), 166.7 (AmidC_q), 194.9 (Thioester-C_q).

Synthesis of Boc-(D)-Thr(tBu)-ABT(Boc):
Following general procedure 1, 551 mg (2.00 mmol) Boc-(D)-Thr(tBu)-OH was reacted with 988 mg (1.90 mmol) PyBOP, 680 µl (4.00 mmol) DIPEA and 621 mg (2.00 mmol) H-ABT(Boc) in 20 ml DMF. Yield: 760 mg (1.34 mmol, 70.5 %), off-white solid. Flash column chromatography: Hexane/EtOAc (6/4, v/v), R_f: 0.45 (Hexane/EtOAc, 1/3, v/v). ^{1}H-NMR (DMSO-d6) δ (ppm): 1.00 (9H, s, *t*Bu), 1.08 (3H, d, CH_3), 1.38 (18H, m, 2*t*Bu), 3.08 (2H, m, N-CH_2-), 3.26 (2H, m, N-CH_2-), 4.07 (4H, m, -S-CH_2, αCH, βCH), 6.49 (1H, d, BocNH), 6.90 (1H, t, BocNH), 7.35 (1H, d, 2ArCH), 7.75 (2H, d, 2ArCH), 8.40 (1H, t, Amid-NH). ^{13}C-NMR (DMSO-d6) δ (ppm): 20.9 (CH_3), 28.6 (2*t*Bu), 28.7 (tBu), 32.3 (-S-CH_2), 66.2 (αCH), 67.4 (βCH_2), 73.9, 78.1, 79.5 (3 C_q), 127.8, 129.1 (2x2ArCH), 133.6, 141.4 (ArC_q), 155.9, 156.2 (2BocC_q), 166.4 (AmidC_q), 201.2 (Thioester-C_q).

Synthesis of H-(D)-Thr-ABT × 2 HCl:
Following general procedure 2, 760 mg (1.33 mmol) Boc-(D)-Thr-ABT(Boc) dissolved in 1.00 ml DCM was treated with 2 ml 4M HCl in Dioxane (abs.) to yield 640 mg (1.66 mmol) H-(D)-Thr-ABT as a colorless solid. ^{1}H-NMR (DMSO-d6) δ (ppm): 1.19 (3H, d, γCH_3), 2.97 (2H, m, N-CH_2-), 3.52 (2H, m, N-CH_2-), 4.15 (3H, m, αCH, βCH_2), 4.29 (2H, m, -S-CH_2), 7.42 (2H, d, 2ArCH), 7.91 (2H, d, 2ArCH), 8.29 (3H, br, NH_3^+), 8.56 (3H, br, NH_3^+), 8.92 (1H, t, AmidNH). ^{13}C-NMR (DMSO-d6) δ (ppm): 20.2 (γCH_3), 32.4 (S-CH_2), 37.6, 39.0 (2N-CH_2-), 64.3, 66.4 (αCH, βCH), 128.2, 129.1 (2x2ArCH), 133.4, 141.0 (2ArC_q), 166.6 (AmidC_q), 195.0 (Thioester-C_q).

Synthesis of Boc-(D)-Tyr(Boc)-ABT(Boc):
Following general procedure 1, 620 mg (1.63 mmol) Boc-(D)-Tyr(tBu)-OH was reacted with 796 mg (1.53 mmol) PyBOP, 548 µl (3.22 mmol) DIPEA and 500 mg (1.61 mmol) H-ABT(Boc) in 16 ml DMF. Yield: 780 mg (1.16 mmol, 76 %), colorless solid. Flash column chromatography: Hexane/EtOAc (2/1, v/v), R_f: 0.62 (Hexane/EtOAc, 1/3, v/v). ^{1}H-NMR (Acetone-d6) δ (ppm): 1.35 (9H, s, *t*Bu), 1.41 (9H, s, *t*Bu), 1.53 (9H, s, *t*Bu), 2.99 (1H, m, βCHH'), 3.26 (H, m, N-CH_2-, βCHH'), 3.49 (2H, m, N-CH_2-), 4.18 (2H, s, -S-CH_2), 4.51 (1H, m, αCH), 6.27 (1H, t, BocNH), 6.70 (1H, d, Boc-NH), 7.10 (2H, d, 2ArCH), 7.31 (2H, d, 2ArCH), 7.42 (2H, d, 2ArCH), 7.85 (3H, m, 2ArCH, Amid-NH). ^{13}C-NMR (Acetone-d6) δ (ppm): 27.6, 27.7 (2*t*Bu), 32.1 (S-CH_2), 36.5 (βCH_2), 62.1

(αCH), 78.0, 79.0, 82.6 ($3C_q$), 121.1, 127.3, 128.7, 130.1 (4x2ArCH), 133.7, 134.8, 141.5, 150.1 ($4ArC_q$), 151.7, 155.1, 155.3 ($3BocC_q$), 166.4 ($AmidC_q$), 200.7 (Thioester-C_q).

Synthesis of H-(D)-Tyr-ABT × 2 HCl:
Following general procedure 2, 410 mg (0.610 mmol) Boc-(D)-Tyr(Boc)-ABT(Boc) dissolved in 1.25 ml DCM was treated with 2.5 ml 4M HCl in Dioxane (abs.) to yield 290 mg (0.600 mmol) H-(D)-Tyr-ABT as a brown foam. ^{1}H-NMR (DMSO-d6) δ (ppm): 2.98 (3H, m, βCHH', N-CH_2-), 3.11 (1, m, βCHH'), 3.54 (2H, m, N-CH_2-), 4.22 (2H, m, -S-CH_2), 4.40 (1H, m, αCH), 6.69 (2H, d, 2ArCH), 6.94 (2H, d, 2ArCH), 7.31 (2H, d, 2ArCH), 7.92 (2H, d, 2ArCH), 8.30 (3H, br, NH_3^+), 8.78 (3H, br, NH_3^+), 8.94 (1H, t, AmidNH). ^{13}C-NMR (DMSO-d6) δ (ppm): 31.9 (S-CH_2), 36.1 (βCH_2), 37.0, 38.5 (2N-CH_2-), 59.4 (αCH), 115.3 (2ArCH), 123.8 (ArC_q), 127.6, 128.6, 130.5 (3x2ArCH), 132.8, 140.3, 156.7 ($3ArC_q$), 166.2 ($AmidC_q$), 195.7 (Thioester-C_q).

Synthesis of Boc-(D)-Val-ABT(Boc):
Following general procedure 1, 940 mg (4.32 mmol) Boc-(D)-Val-OH was reacted with 2.14 mg (4.11 mmol) PyBOP, 1.48 ml (8.65 mmol) DIPEA and 1.34 g (4.32 mmol) H-ABT(Boc) in 43 ml DMF. Yield: 1.79 g (3.51 mmol, 85.5 %), colorless foam. Flash column chromatography: Hexane/EtOAc (3/1, v/v), R_f: 0.60 (Hexane/EtOAc, 1/3, v/v). ^{1}H-NMR ($CDCl_3$) δ (ppm): 0.84 (γCH_3), 0.97 (γCH_3), 1.45 (18H, m, 2*t*Bu), 2.78 (1H, m, βCH), 3.40 (2H, m, N-CH_2-), 3.55 (2H, m, N-CH_2-), 4.13 (2H, m, -S-CH_2), 4.30 (1H, m, αCH), 4.99 (1H, d, BocNH), 5.10 (1H, br, BocNH), 7.33 (2H, d, 2ArCH), 7.75 (2H, d, 2ArCH). ^{13}C-NMR ($CDCl_3$) δ (ppm): 16.8, 19.4 ($2\gamma CH_3$), 28.3 (2*t*Bu), 31.0 (βCH), 32.8 (-S-CH_2), 40.0, 42.1 (2N-CH_2-), 65.2 (αCH), 80.1, 80.3 ($2C_q$), 127.4, 128.9 (2x2ArCH), 133.0, 141.2 (ArC_q), 155.5, 157.6 ($2BocC_q$), 167.5 ($AmidC_q$), 200.4 (Thioester-C_q).

Synthesis of H-(D)-Val-ABT × 2 HCl:
Following general procedure 2, 1.79 g (3.50 mmol) Boc-(D)-Val-ABT(Boc) dissolved in 7.00 ml DCM was treated with 14 ml 4M HCl in Dioxane (abs.) to yield 1.33 g (3.48 mmol) H-(D)-Val-ABT as an off-white foam. ^{1}H-NMR (DMSO-d6) δ (ppm): 0.92 (6H, m, $2\gamma CH_3$), 2.19 (1H, m, βCH), 2.97 (2H, m, N-CH_2-), 3.53 (2H, m, N-CH_2-), 4.13 (1H, m, αCH), 4.30 (2H, m, -S-CH_2), 7.41 (2H, d, 2ArCH), 7.92 (2H, d, 2ArCH), 8.34 (3H, br, NH_3^+), 8.73 (3H, br, NH_3^+), 8.94 (1H, t, AmidNH). ^{13}C-NMR (DMSO-d6) δ (ppm): 18.5, 18.1 ($2\gamma CH_3$), 30.7 (βCH), 32.5 (S-CH_2), 37.6, 38.9 (2N-CH_2-), 63.6 (αCH), 128.2, 129.1 (2x2ArCH), 133.4, 141.1 ($2ArC_q$), 166.6 ($AmidC_q$), 195.9 (Thioester-C_q).

Synthesis of (L)-aa Flexizyme substrates
(L)-Phe-CME, (L)-Trp-CME and (L)-Tyr-CME were purchased from IRIS Biotechnology (Marktredwitz, D). (L)-Ala-ABT, (L)-Ser-ABT and (L)-Met-DBE were synthesized as described above for (D)-Ala-ABT, (D)-Ser-ABT and (D)-Met-DBE, respectively. The synthesis of (L)-Asn-ABT, (L)-Asp-ABT, (L)-Arg-ABT, (L)-Cys-ABT, (L)-Gln-ABT, Gly-ABT, (L)-His-ABT, (L)-Ile-ABT, (L)-Leu-ABT, (L)-Lys-ABT, (L)-Pro-ABT, (L)-Thr-ABT, (L)-Val-ABT was commenced from Boc- and eventually side-chain protected (L)-aa-OSu esters, the protection pattern was the same as given for the corresponding D-aa. L-aa-OSu esters were dissolved in DMF and reacted with 0.85 eq. H-ABT in presence of 3 eq. DIPEA and N-methylimidazole or DMAP. Purification and final acidic deprotection was performed as described above for the corresponding D-aa-ABT. The physical properties (NMR) of the final products were identical with the corresponding D-aa-ABT thioesters.

3'-^{32}P-Radiolabeling of tRNA
In presence of pyrophosphate, tRNA nucleotidyltransferase (TRNT) truncates a tRNA from the 3' end by two nucleotides. The equilibrium of the reaction is then shifted by degradation of pyrophosphate using inorganic pyrophosphatase and addition of CTP. The reactions were assembled as follows: 5 µl of 1M glycine pH 9, 1 µl of 1M $MgCl_2$, 0.5 µl of 10 mM pyrophosphate, 9 µl of α-[^{32}P]-ATP (3,000 Ci/mmol; Hartmann Analytics), 6.25 µl of 400 µM tRNA, 3 µl of 6.7 µg/µl TRNT and H_2O to 100 µl were mixed and incubated at 37°C for three minutes for truncation. 2 µl of 9 µg/µl inorganic pyrophosphatase from *E. coli* and 0.9 µl of 100 µl CTP were

then added and incubation at 37°C was continued for another 2 minutes. Radiolabeled tRNA was purified immediately using the NucleoSpin RNA clean-up XS kit (Macherey-Nagel). To allow for purification of small RNAs, the supplied binding buffer was replaced by 0.1 volumes of 3M NaOAc pH 5.5 (Ambion) and 3 volumes of isopropanol. The tRNA was eluted twice with 30 µl of RNase-free water.

Misacylation of tRNA

For a typical reaction for downstream use in translation experiments, 10 µl of 500 mM HEPES-KOH pH 7.5, 3.75 µl of 1 mM Flexizyme and 2.5 µl of 1 mM tRNA and H_2O to 60 µl were heated to 95°C for 2 min. and cooled to 20°C at a rate of 0.2°C/s. 20 µl of 3 M $MgCl_2$ were added, the reaction was incubated for 5 min. at room temperature and 2 min. on ice, then 20 µl of 25 mM Flexizyme substrate in DMSO were added and incubated for 3 h on ice. The RNA was precipitated by the addition of 0.1 volumes of 3 M NaOAc pH 5.5 (Ambion), 2.5 volumes of room temperature ethanol and centrifugation for 30 minutes at 21,500 x g and 25°C (to prevent co-precipitation of magnesium). The pellets were washed with 300 mM NaOAc pH 5.5 in 70% ethanol, again with 70% ethanol, air-dried, dissolved in 5 µl of H_2O, and stored at -80°C until use within the same week.

For downstream use in EF-Tu electrophoretic mobility shift assays, tRNA was misacylated in batch for each series of experiments. The reaction volume per subsequent assay was scaled down to 20 µl and ≈400,000 cpm of 3'-^{32}P-radiolabeled tRNA were used instead of non-labeled tRNA. Misacylated tRNA was dissolved in 3 µl of H_2O per subsequent assay and stored in aliquots of 3 µl at -80°C until use within the same week. One aliquot was generally used to determine the aminoacylation ratio following an earlier protocol (13): to one aliquot of 3 µl, 1 µl of S1 nuclease and 1 µl of 5x S1 nuclease buffer (Fermentas) were added and digestion was allowed to proceed for 10 min. at 37°C. The digests contained ^{32}P-AMP and aminoacyl-^{32}P-AMP, which were separated by thin layer chromatography (TLC) on polyethyleneimine-cellulose (Macherey-Nagel), the mobile phase was 5% acetic acid (v/v) with 100 mM NH_4Cl. K-Screens (Kodak) were exposed to the TLC-sheets usually for 2-4 hours (as appropriate to obtain clear signals without saturated pixels) at -80°C and scanned with a Molecular Imager FX (Bio-Rad) at 50 µm resolution (508 dpi). The spots of aminoacyl-^{32}P-AMP and ^{32}P-AMP were quantified using ImageLab software (Bio-Rad) and the aminoacylation ratio was calculated as [aminoacyl-^{32}P-AMP / (aminoacyl-^{32}P-AMP + ^{32}P-AMP)].

EF-Tu electrophoretic mobility shift assay (EMSA)

To set the GTP state, a mastermix containing per reaction 3 µl of 16.67 µM EF-Tu in EF-Tu storage buffer (50 mM HEPES-KOH pH 7.6 @ 0°C, 50 mM KCl, 1 mM DTT, 10% glycerol) and 2 µl of 100 mM HEPES-KOH pH 7.6 @ 0°C, 130 mM NH_4OAc; 20 mM $Mg(OAc)_2$, 25 mM phosphoenol pyruvate, 2.5 mM GTP, 500 µM DTT, 0.2 mg/mL pyruvate kinase from rabbit muscle (Sigma) was incubated for 15 min. at 37°C to set the GTP state. 2 µl per reaction of ternary complex buffer [150 mM HEPES-KOH pH 7.6 @ 0°C; 195 mM NH_4OAc; 30 mM $Mg(OAc)_2$] were added and the mix was spread into reaction vessels containing 3 µl of radiolabeled aminoacyl-tRNA or deacyl-tRNA in H_2O. Incubation was continued at 37°C for 10 min. 2 µl of 50% glycerol with bromophenol blue were added and the samples applied to native 8% polyacrylamide gels (acrylamide:bisacrylamide 19:1, dimensions 18×18 cm) that were cast in 1x running buffer. The running buffer was 10 mM MES-NH_4OH pH 6.7; 10 mM $Mg(OAc)_2$; 65 mM NH_4OAc; 1 mM Na-EDTA; 1 mM DTT, and 10 µM GTP. To reduce band smearing, samples were loaded under applied voltage. Gels were run for 220 min. at 150 V. K-Screens (Kodak) were exposed to the gels for 4 hours (for quantification purposes) or overnight (for qualitative analyses) at -80°C and scanned with a Molecular Imager FX (Bio-Rad) at 50 µm resolution (508 dpi). For evaluation using ImageLab software (Bio-Rad), three volumes per lane were defined: One that includes the ternary complex band, one that includes the free tRNA including the area between the distinct bands (thus including any smear

due to TCs dissociated during gel electrophoresis) and one right above the TC band that defines the background for that lane.

Activity test of EF-Tu mutants by GFP translation

A mastermix was prepared comprising per reaction H_2O to a final reaction volume of 20 µl, 2 µl of SUPERaseIn (Ambion), 2 µl of 120 mM $Mg(OAc)_2$, 2 µl Solution 1, 1.2 µl Solution 2 (all canonical amino acids), 1 µl Solution 3, 1 µl Solution 4 (all aaRS), and 1 µl of 200 ng/µl **pRSET/EmGFP** (Intitrogen). The mastermix was spread to white twin.tec 96-well real-time PCR plates (Eppendorf). 2 µl of 2,000 ng/µl wild-type-EF-Tu or mutant EF-Tu in EF-Tu storage buffer (see above) were added and mixed by pipetting. The plates were sealed and incubated at 37°C in a PolarStar Optima microplate reader (BMG lab technologies). Fluorescence was measured by excitation at 485 nm and detection at 520 nm wavelength every ten min. until it reached a plateau.

Translation and detection of ^{35}S-Met labelled peptides

For a typical experiment, reaction vessels were prepared containing either 1 µl of H_2O and 1 µl of 500 µM preacylated tRNA or for control reactions 1 µl of 500 µM deacyl-tRNA and 1 µl of the respective L-aa-tRNA diluted 1:100 in H_2O. A mastermix comprising per reaction H_20 to a final reaction volume of 10 µl, 1 µl of SUPERaseIn (Ambion), 1 µl of 120 mM $Mg(OAc)_2$, 1 µl of solution 1, 0.6 µl of solution 2 (devoid of ^{32}S-Met and unnecessary amino acids), 0.5 µl each of solutions 3 and 4 (devoid of unnecessary aaRS), 1 µl of 2,000 ng/µl EF-Tu (in EF-Tu storage buffer, see above), 0.5 µl of 200 ng/µl template and 0.8 µl of ^{35}S-Met (SCM-01; Hartmann Analytics) was spread to the prepared reaction vessels. The final magnesium concentration is 13 mM (1 mM from solutions 3 and 4). The reactions were incubated for 3 h at 37°C. For double and triple incorporation experiments, the final EF-Tu concentration was doubled and EF4 was added to 0.36 µM (0.3 ribosome equivalents). For purification of the translated peptides, 20 µl of Ni-NTA-Agarose (Qiagen) were spun dry in MoBiSpin F columns (MoBiTec) and equilibrated with 200 µl of 50 mM Tris-HCl pH 7.5, 500 mM NaCl, 20 mM imidazole. 90 µl of the same buffer and one reaction of 10 µl were added and shaken at 750 rpm at 4°C for 1 h. The columns were spun dry, washed with 200 µl of water, and eluted twice with 100 µl each of 10% formic acid. The eluates were dried in a SpeedVac and subjected to 16% TRICINE-SDS PAGE (2 h at 125 V, gels and buffers from novex). Phosphorimager-Screens (K-Screens, Kodak) were exposed for 3-4 days and scanned using a MolecularImager FX (Bio-Rad) at 50 µm resolution (508 dpi).

For relative quantification of bands using ImageLab software (Bio-Rad), free-hand volumes were defined precisely around the bands and background was subtracted globally. Care was taken not to underestimate bands of L-aa or control reactions as well as not to overestimate bands of D-aa reactions.

Diastereomeric peptide separation method and mass spectrometric detection.

The RP-HPLC method for separation of the D- and L-peptide isomers was established on synthetic peptides (formyl-MSKAKFARTKPHANA[(D-W or L-W)]HHHHHH), monoisotopic mass 2693.3 Da; Biosyntan). Furthermore, these peptides were used for translation sample spiking as well as a standard mixture for LC-MS performance control before and after sample analysis. As an internal standard for the entire LC-MS performance, the synthetic tripeptide VYV (Sigma-Aldrich, MW 379.2Da, detectable in a single charge state of $M+H^+$ with 380.2 *m/z*) was added to each sample. Separation has been achieved using an Agilent 1290 chromatographic system (Agilent Technologies) and accurate mass analysis using an online coupled ESI – QTOF 6520 (Agilent Technologies) mass spectrometer. Samples were injected into an Acquity UPLC BEH300 C18 column (2.1 x 100mm, 1.7µm particle size, 300Å pore size; Waters) and desalted for 0.7 minutes before switching the flow online to the MS. Elution was performed with a gradient of solvent A and B in 30 minute runs: after 3 min. of desalting with 100% solvent A, separation was performed with 0-15% solvent B in 17 min. followed by a column wash with 15-80% solvent B in 1 min. 80% solvent B was kept for 2 min. to elute the

column completely. Solvent A was increased to 100% in 1 min. and the column was re-equilibrated for 6 min. Solvent A consisted of 0.5% acetonitrile and 0.2% formic acid in water and solvent B consisted of 4.8% water and 0.2% formic acid in acetonitrile. Column temperature was set to 60°C and flow kept constant at 0.4 ml/min. TOF-MS was acquired in high resolution positive mode (mass range of 300 to 3200 *m/z*, with a scan time of 500 ms/spectrum). Expected full length peptides were detected by generating merged Extracted Ion Chromatograms (EIC) of the most intense charge states of the full length peptides [$(M+5H)^{5+}$: 539.6 *m/z*, $(M+4H)^{4+}$: 674.3 *m/z*, $(M+3H)^{3+}$: 898.7 *m/z*) and of the internal standard tripeptide VYV (380.2 *m/z*) with asymmetric peak detection (+ 0.7 Da / -0.3 Da).

Translation and peptide detection by LC-MS. Template 'G_1' was translated in presence of 50 µM deacyl-$tRNA^{Gly}_u$ (negative control), 50 µM L-Trp-$tRNA^{Gly}_u$ or 50 µM D-Trp-$tRNA^{Gly}_u$. Translation reactions were scaled up to 100 µl for the negative control and 300 µl for the L-Trp/D-Trp reactions as given above. Final EF-Tu concentration was 400 ng/µl, EF4 was added to 0.36 µM (0.3 ribosome equivalents) and the reactions contained 0.3 mM of ^{32}S-Met instead of ^{35}S-Met. Purification was done as described above. Dried samples were dissolved in Solvent A. Products from L-Trp and D-Trp reactions were split into three equal parts, one part was spiked with 50 ng of the synthetic L-Trp containing peptide and one part was spiked with 50 ng of the synthetic D-Trp containing peptides (Biosyntan). All samples were spiked with 50 ng of the synthetic tripeptide VYV (Sigma) and analysed as described above. The truncated peptide (formyl-MSKAKFARTKPHANA, monoisotopic mass 1684.8Da) was detected by the triply charged ion ($(M+3H)^{3+}$: 562.6 *m/z*). Peptides containing L-Ile, D-Ile, L-Val or D-Val were translated in 100 µl reactions and purified as given above. Minor changes in the LC-MS analysis procedure: the column temperature was set to 30°C and merged Extracted Ion Chromatograms were generated with symmetric peak detection ±10 ppm.

Supporting References

1. Shimizu, Y. and Ueda, T. (2010) PURE technology. *Methods Mol Biol*, **607**, 11-21.
2. Effraim, P.R., Wang, J., Englander, M.T., Avins, J., Leyh, T.S., Gonzalez, R.L., Jr. and Cornish, V.W. (2009) Natural amino acids do not require their native tRNAs for efficient selection by the ribosome. *Nat Chem Biol*, **5**, 947-953.
3. Beaucage, S.L. and Caruthers, M.H. (1981) Deoxynucleoside phosphoramidites—A new class of key intermediates for deoxypolynucleotide synthesis. *Tetrahedron Letters*, **22**, 1859-1862.
4. Scaringe, S.A., Francklyn, C. and Usman, N. (1990) Chemical synthesis of biologically active oligoribonucleotides using beta-cyanoethyl protected ribonucleoside phosphoramidites. *Nucleic Acids Res*, **18**, 5433-5441.
5. Wincott, F., DiRenzo, A., Shaffer, C., Grimm, S., Tracz, D., Workman, C., Sweedler, D., Gonzalez, C., Scaringe, S. and Usman, N. (1995) Synthesis, deprotection, analysis and purification of RNA and ribozymes. *Nucleic Acids Res*, **23**, 2677-2684.
6. Goto, Y., Katoh, T. and Suga, H. (2011) Flexizymes for genetic code reprogramming. *Nat Protoc*, **6**, 779-790.
7. Itoh, M., Hagiwara, D. and Kamiya, T. (1975) A new tert-butyloxycarbonylating reagent, 2-tert-butyloxycarbonyloxyimino-2-phenylacetonitrile. *Tetrahedron Letters*, **16**, 4393-4394.
8. Pozdnev, V.F. (1982) tert-Butoxycarbonylation of tyrosine and other phenolic amino acids with di-tert-butyl pyrocarbonate. *Chemistry of Natural Compounds*, **18**, 125-126.
9. Keller, O., Keller, W.E., Look, G.v. and Wersin, G. (2003), *Organic Syntheses*. John Wiley & Sons, Inc.
10. Nagalingam, A.C., Radford, S.E. and Warriner, S.L. (2007) Avoidance of Epimerization in the Synthesis of Peptide Thioesters Using Fmoc Protection. *Synlett*, **2007**, 2517-2520.
11. Coste, J., Le-Nguyen, D. and Castro, B. (1990) PyBOP®: A new peptide coupling reagent devoid of toxic by-product. *Tetrahedron Letters*, **31**, 205-208.
12. Wang, W. and McMurray, J.S. (1999) A selective method for the preparation of primary amides: Synthesis of Fmoc-l-4-carboxamidophenylalanine and other compounds. *Tetrahedron Letters*, **40**, 2501-2504.

13. Ledoux, S. and Uhlenbeck, O.C. (2008) [3'-32P]-labeling tRNA with nucleotidyltransferase for assaying aminoacylation and peptide bond formation. *Methods*, **44**, 74-80.
